# *In vitro* characterization of hemoglobin oxygen dissociation curves and electrolyte shifts in human blood under varying PCO_2_

**DOI:** 10.3389/fmed.2025.1708274

**Published:** 2026-01-12

**Authors:** Carlo Valsecchi, Eleonora Carlesso, Michele Battistin, Sebastiano M. Colombo, Emanuele Cattaneo, Francesca Gori, Thomas Langer, Giacomo Grasselli, Alberto Zanella

**Affiliations:** 1 Department of Anesthesia, Critical Care and Emergency, Fondazione IRCCS Ca’ Granda-Ospedale Maggiore Policlinico, Milan, Italy; 2 Department of Pathophysiology and Transplantation, University of Milan, Milan, Italy; 3 Center for Preclinical Research, Fondazione IRCCS Ca’ Granda Ospedale Maggiore Policlinico, Milan, Italy; 4 Department of Medicine and Surgery, University of Milano-Bicocca, Monza, Italy; 5 Department of Anesthesia and Intensive Care Medicine, Niguarda Ca’ Granda, Milan, Italy

**Keywords:** hemoglobin, oxygen, carbon dioxide, electrolytes, physiology, blood, erythrocyte

## Abstract

**Background:**

Efficient oxygen transport depends on hemoglobin (Hb) affinity for O_2_, which is modulated by factors like PCO_2_, as described by the Bohr effect. This *in vitro* study explored how varying PO_2_ and PCO_2_ influence hemoglobin oxygen saturation (HbO_2_) and plasma electrolyte concentrations in whole human blood.

**Methods:**

Blood from six healthy volunteers was equilibrated at 37°C with gas mixtures spanning PO_2_ and PCO_2_ ranges. A total of 346 samples were analyzed for blood gases, HbO_2_, and electrolytes. The HbO_2_ dissociation curve was modeled using a Gompertz function within a non-linear mixed-effects framework, while electrolyte dynamics were assessed via polynomial models.

**Results:**

HbO_2_ saturation ranged from 1.4 to 99.6%. Increasing PCO_2_ shifted the dissociation curve rightward, steepening its slope and raising the inflection point—hallmarks of the Bohr effect—without affecting maximal HbO_2_. Electrolyte analysis revealed that chloride decreased with PCO_2_ and increased with HbO_2_, consistent with the erythrocyte chloride shift. Sodium increased with PCO_2_, and a significant interaction between HbO_2_ and PCO_2_ was observed. Strong ion difference (SID) decreased linearly with HbO_2_ and increased quadratically with PCO_2_, suggesting a compensatory role in CO_2_-induced acid-base changes.

**Conclusion:**

These findings, validated against external datasets, underscore the tight coupling between respiratory gas exchange and electrolyte homeostasis. The study provides novel insights into how CO_2_ modulates both oxygen delivery and plasma ionic composition, with implications for understanding acid-base physiology and its regulation in health and disease.

## 1 Introduction

The efficient transport of oxygen (O_2_) in mammals is a critical physiological process that underpins cellular respiration and metabolic function. If O_2_ transport relied solely on O_2_ dissolved in plasma, i.e., on Henry’s law, mammals would require an increased atmospheric pressure (1) to maintain an adequate O_2_ delivery to the tissues. Alternatively, to meet basal metabolic demands without hemoglobin (Hb), the cardiac output would need to rise to nearly 100 liters per minute—far beyond physiological limits.

Evolution has equipped mammals and nearly all vertebrates with Hb, a globular protein that facilitates oxygen transport. Hb reversibly binds O_2_, enabling efficient delivery from high-pressure environments (lungs) to low-pressure areas (tissues) where oxygen is utilized by mitochondria.

Hb is the second most abundant protein in the human body after collagen, comprised of four subunits—two alpha and two non-alpha chains—forming a tetrameric structure. Each subunit is associated with a heme group, which contains an iron ion (Fe^2 +^) that is essential for oxygen binding. The cooperative nature of O_2_ binding to Hb leads to the characteristic sigmoidal oxygen dissociation curve, a phenomenon first described by Paul Bert (2) and later explained in greater detail by Bohr (3–5) who highlighted the relationship between pH, partial pressure of carbon dioxide (PCO_2_), and the affinity of Hb for O_2_. The so called “Bohr effect” elucidates how increasing PCO_2_ and proton concentration (H^+^) reduces Hb’s affinity for O_2_, promoting its release in metabolically active tissues. Conversely, the Haldane effect (6, 7) describes how deoxygenated Hb can bind CO_2_ more effectively, enhancing the transport of CO_2_ to the lungs for elimination. This interplay between O_2_ and CO_2_ binding is crucial for maintaining acid-base balance and ensuring efficient gas exchange.

The conformational changes of Hb are crucial for gas exchange: the transition from a relaxed (R) state, characterized by high affinity for O_2_, to a taut (T) state, with lower affinity for O_2_, facilitates the transport and the subsequent release of O_2_ and CO_2_ between peripheral tissues and the lungs, and vice versa. Factors such as temperature, pH, and the presence of various ligands (e.g., 2,3-diphosphoglycerate (2,3-DPG), chloride ions) can modulate Hb conformational changes.

The transition from the R to the T conformation also enables Hb to bind or release other molecules, including chloride ions (Cl^–^) and protons (H^+^) (8, 9). More broadly, the blood flowing through systemic capillaries, and the resulting changes in gas partial pressures and electrolyte concentrations, leads to a net movement of charges and water across the erythrocyte membrane, and a shift in intracellular charge distribution (10).

Understanding these interactions is essential to understand how Hb responds to varying physiological and pathological conditions, allowing for optimized gas exchange, even under extreme circumstances.

The primary aim of our study is to investigate the behavior of O_2_ binding to Hb at varying partial pressures of O_2_ (PO_2_) and of CO_2_ (PCO_2_) in human whole blood and to explore how these variations influence plasma electrolyte levels. While the oxygen dissociation curve and hemoglobin conformational changes have been extensively characterized, the novel contribution of this work lies in the analysis of plasma electrolyte variations in response to changes in gas partial pressures. This aspect has received limited attention in previous literature, despite its physiological relevance. By coupling gas exchange dynamics with electrolyte behavior, our study may reveal previously unrecognized interactions between respiratory gases and membrane ion transport. This research is significant not only for advancing our understanding of the fundamental principles in respiratory physiology, but also for its potential clinical implications, particularly in conditions that affect acid-base balance, oxygen delivery, and carbon dioxide transport. By shedding light on the intricate dynamics of Hb function, we aim to contribute to the broader understanding of respiratory physiology and its critical role in maintaining homeostasis in the human body.

## 2 Materials and methods

The present study received approval from the Ethical Committee of Fondazione Ca’ Granda Ospedale Maggiore Policlinico (Milan) (Identifier: 429_2019; 21st May 2019). Six healthy volunteers were included in the study following the acquisition of written informed consent.

### 2.1 Blood sample collection

A total of 42 ml of venous blood was collected from each subject via peripheral venipuncture and distributed in 10 tubes.

Seven lithium-heparin tubes (Vacuette^®^ Plasma Lithium Heparin, 4 mL, Greiner Bio-One™, Kremsmünster, Austria) were used for tonometry. The remaining three samples—two EDTA tubes (3 ml) and one K2EDTA tube with gel (3.5 mL)—were sent for complete blood count, electrolytes, albumin, total protein, and Hb electrophoresis, analyzed using the Cobas^®^ 8000 modular analyzer (Roche, Basel, Switzerland).

### 2.2 Sample preparation and PCO_2_ tonometry

Whole blood anticoagulated with lithium heparin was stored at 6°C for at least 30 min to standardize samples and minimize 2,3-DPG degradation (no-touch phase). Each sample was then aspirated into a syringe pre-treated with anti-foam concentrate (T310, RNA Medical, Devens, MA, United States). Blood samples were equilibrated at 37°C with gas mixtures of known PO_2_ and PCO_2_, using a tonometer (Equilibrator, RNA Medical, Devens, MA, United States). Complete O_2_ saturation of hemoglobin at the target CO_2_ levels (10, 20, 50, 70, or 90 mmHg) was obtained by equilibrating the blood in a continuous-bubble tonometer with a gas mixture of known PO_2_ and PCO_2_. After verifying full hemoglobin saturation by blood gas analysis (blood PO_2_ > 600 mmHg), Hb desaturation was titrated by equilibrating the sample with a gas mixture containing the same target PCO_2_ (10, 20, 50, 70, or 90 mmHg), a PO_2_ of zero, and a nitrogen pressure (PN_2_) adjusted to achieve a total pressure (Ptot) equal to atmospheric pressure (Patm). The titration proceeded until either a PO_2_ < 10 mmHg was reached or lactate levels increased by more than 2 mmol⋅L^–1^; relative to baseline. Samples with lactate increases more than 2 mmol/L from baseline were excluded.

### 2.3 Blood gas analysis

During deoxygenation, samples were repeatedly analyzed at 37°C using a blood gas and electrolyte analyzer (ABL90 FLEX, Radiometer, Copenhagen, Denmark). Measured values were recorded in an Excel spreadsheet for further analysis.

Strong ion difference (SID) was calculated as follows:


$$\left[SID\right]=\left[Na^{+}\right]+\left[K^{+}\right]+\left[Ca^{2+}\right]-\left[Cl^{-}\right]-\left[Lac^{-}\right]$$

where [Na^+^], [K^+^], [Ca^2 +^], [Cl^–^], and [Lac^–^] refer to the plasma concentrations of sodium, potassium, calcium, chloride, and lactate, respectively, expressed in mEq/L as measured by the blood gas analyzer. Magnesium [Mg^2+^] and Phosphate [PO_4_^3–^] were excluded due to lack of measurement by the blood gas analyzer and minimal impact on SID.

### 2.4 Mathematical models and statistical analysis

#### 2.4.1 Modeling the hemoglobin-oxygen curve at different PCO_2_ levels

In this study, we modeled the hemoglobin-oxygen dissociation curve using a three-parameter Gompertz function, which is well-suited for capturing sigmoidal shapes due to its flexibility. A mixed-effects Gompertz model was implemented using SAS PROC NLMIXED to assess the influence of PCO_2_ and random effects.

The Gompertz function used is defined as:


$$HbO_{2}=a\cdot e^{-e^{\frac{-\left(PO_{2}-x0\right)}{b}}}$$

Where:

1. **a** represents the upper asymptote of the curve.
2. **x0** represents the inflection point of the curve along the *x*-axis.
3. **b** controls the growth rate and the steepness of the curve.

To evaluate parameter distributions and determine which should be modeled as fixed or random effects, we conducted preliminary analyses on individual subject data. Further details are provided in Supplementary material.

##### 2.4.1.1 Building the model

The nonlinear mixed model was initially constructed without random effects, modeling each parameter (a, b, x0) as a linear function of PCO_2_. Residual variance (s2e) was included. The equations were:


$$a=afix+aPCO_{2}\cdot PCO_{2}$$

$$b=bfix+bPCO_{2}\cdot PCO_{2}$$

$$x0=x0fix+x0PCO_{2}\cdot PCO_{2}$$

Random effects were added stepwise: -individually, in pairs, and jointly-. Effects were retained in the model only if significant (*P* ≤ 0.05). PCO_2_ was treated as a continuous variable. Initial values for fixed components were derived from individual fits. Full model selection details are provided in Supplementary material. To better understand how PCO_2_ affects oxygen affinity, we derived P50 values from the Gompertz model. P50 represents the PO_2_ at which hemoglobin is 50% saturated and is widely used to describe shifts in the oxygen–hemoglobin dissociation curve.

#### 2.4.2 Strong ion difference and electrolytes

We investigated the effects of HbO_2_ and PCO_2_ on strong ion difference (SID) and electrolyte concentrations using polynomial multilevel models with both fixed and random effects.

To assess potential collinearity among the independent variables (HbO_2_ and PCO_2_), we calculated the variance inflation factor (VIF) and performed collinearity diagnostics, including the evaluation of eigenvalues and condition indices.

As an initial exploratory step, scatter plots with univariate regressions were used to examine the relationships between the dependent variables (SID and electrolytes) and the independent variables HbO_2_ and PCO_2_.

We then modeled the effects of HbO_2_ and PCO_2_ on the dependent variables, using a polynomial multilevel model with fixed and random effects, treating both variables as continuous. The inclusion of new effects and the functional form of the relationships were evaluated using likelihood ratio tests (significance threshold: *P* ≤ 0.100). The optimal covariance structure for the mixed model was selected based on the Akaike Information Criterion (AIC), ensuring the best fit to the data. Model adequacy was assessed through residual analysis, and model validation was performed using bootstrap resampling (see Supplementary material for further details).

The SID and electrolytes models were then tested using experimental data from 18 healthy subjects, as reported by Langer et al. (11).

Values of SID, Na^+^ and Cl^–^ were estimated using our models at the average PCO_2_ values reported in the Langer’s study, assuming HbO_2_ of 98% as the original paper did not report HbO_2_ values. The authors stated that venous blood samples were tonometrically oxygenated at 21% O_2_. Based on this, we assumed that the blood was fully oxygenated at the end of the tonometry process. Since HbO_2_ is typically slightly lower than oxygen saturation due to the presence of carboxyhemoglobin and methemoglobin, we adopted a physiologically plausible HbO_2_ value of 98% for our estimations.

#### 2.4.3 Other statistical analysis

Continuous data are presented as median and interquartile range (IQR) or mean [ ± standard deviation, SD], as appropriate. We did not apply any imputation for missing values. The initial assessment of the relationships between electrolytes, HbO_2_ and PCO_2_, between the values of PO_2_ at which Hb is 50% saturated (P50) and PCO_2_ and between SID and electrolytes were modeled by univariate regressions.

Analyses were performed using SAS 9.4 (SAS Institute, Cary, North Carolina, United States) and SigmaPlot (Systat Software, San Jose, CA).

## 3 Results

Table 1 summarizes the general characteristics of the population and presents the results of the blood analyses. Of note all parameters are in the normal range. No Hb alterations were detected.

**TABLE 1:** Characteristics of population, blood count and electrolytes concentrations prior to any manipulation.

Variables	Median [IQR]
Age (years)	31 [29–34]
Height (m)	1.78 [1.75–1.81]
Weight (kg)	71.5 [70.3–75.0]
BMI (kg/m^2^)	23.03 [22.27–23.50]
Sodium (mmol/L)	142 [141–143]
Potassium (mmol/L)	4.35 [4.15–4.55]
Chlorine (mmol/L)	102 [101–103]
Tot. Protein (g/dL)	7.20 [7.03–7.30]
Albumin (g/dL)	4.8 [4.6–4.8]
Calcium (mg/dL)	9.55 [9.42–9.61]
Phosphate (mg/dL)	3.2 [3.1–3.7]
Magnesium (mg/dL)	2.10 [2.02–2.14]
White blood cells (10^9/L)	5.49 [5.29–6.47]
Red blood cells (10^9/L)	5.32 [4.98–5.43]
Hemoglobin (g/dL)	15 [15–16]
Hematocrit (%)	43.7 [42.1–44.5]
Mean globular volume (fL)	82.6 [80.9–84.8]
Anisocytosis index (%)	35.1 [34.4–35.3]
MCH (pg)	12.3 [11.9–12.7]
MCHC (g/dL)	29.1 [27.9–29.9]
HbA2 (%)	35.1 [34.4–35.3]
HbF (%)	2.7 [2.6–2.8]

(MCHC, Mean Corpuscular Hemoglobin Concentration; MCH, Mean Corpuscular Hemoglobin; HbA2, A2 Hemoglobin; HbF, Fetal Hemoglobin).

Six healthy male subjects aged between 28 and 36 years old (mean age, 32 ± 3 years) were enrolled in the study. A total of 361 blood samples were collected, of which 15 (4.2%) were excluded from statistical analysis due to a lactate increase > 2 mEq/L compared to baseline. The final dataset therefore included 346 samples, with a median lactate variation of 0.3 mEq/L (IQR 0.1–0.6 mEq/L).

In the 346 blood samples titrated with nominal PCO_2_, the measured PCO_2_ values ranged between 6 and 111 mmHg (median 52.6 mmHg, IQR 18.1–73.7) and measured PO_2_ ranged between 0.1 and 721.0 mmHg (median 57.6 mmHg, IQR 32.5–97.5), resulting in a range of HbO_2_ from 1.4 to 99.6% (median 89.8%, IQR 67.2–96.7%).

### 3.1 Oxygen–hemoglobin dissociation curve

#### 3.1.1 Analysis of experimental data and individual curve fitting

The results of the preliminary data analyses, including experimental data, individual fits, residuals, parameter distributions, and model selection details, are provided in Supplementary material. The inspection of the residuals indicated that the Gompertz model adequately described our data.

Graphs of the individual parameter means and 95% CIs at different nominal PCO_2_ levels suggested the need to test whether the parameters should be treated as random or fixed effects. The upper asymptote **a** is not affected by PCO_2_, on the contrary **b** and **x0** are right-shifted at increasing nominal PCO_2_ values (Supplementary Table 5).

#### 3.1.2 Building the mixed model

Details of the parameter selection process are provided in Supplementary material. The analysis showed that random effects did not significantly improve the model, so they were excluded. As expected from the individual curve fittings, parameter **a** did not show a significant dependence on PCO_2_ (**a**_*PCO*2_: *P* = 0.6751) and was therefore excluded from the model. Consequently, the final model was constructed without random effects with PCO_2_ affecting parameters **b** and **x0:**


$$HbO_{2}=a\cdot e^{-e^{\frac{-\left(PO_{2}-x0\right)}{b}}}$$

where:


$$b=bfix+bPCO_{2}\cdot PCO_{2}$$

$$x0=x0fix+x0PCO_{2}\cdot PCO_{2}$$

Table 2 reports the estimated parameters, along with standard errors, CIs and *P*-values.

**TABLE 2:** Estimated parameters from the nonlinear mixed model (Gompertz equation) applied to describe oxygen-hemoglobin dissociation curves at different PCO_2_.

Parameters	Estimate	Standard error	95% CIs		*P*-value
a	97.1399	0.1548	96.8354	97.4444	<0.0001
bfix	9.2308	0.2089	8.8199	9.6418	<0.0001
bPCO_2_	0.1062	0.0037	0.0989	0.1136	<0.0001
x0fix	11.1305	0.1659	10.8043	11.4568	<0.0001
x0PCO_2_	0.1793	0.0031	0.1732	0.1855	<0.0001
s2e	3.2723	0.2488	2.7830	3.7616	<0.0001

CIs, confidence limits.

**FIGURE 1:**
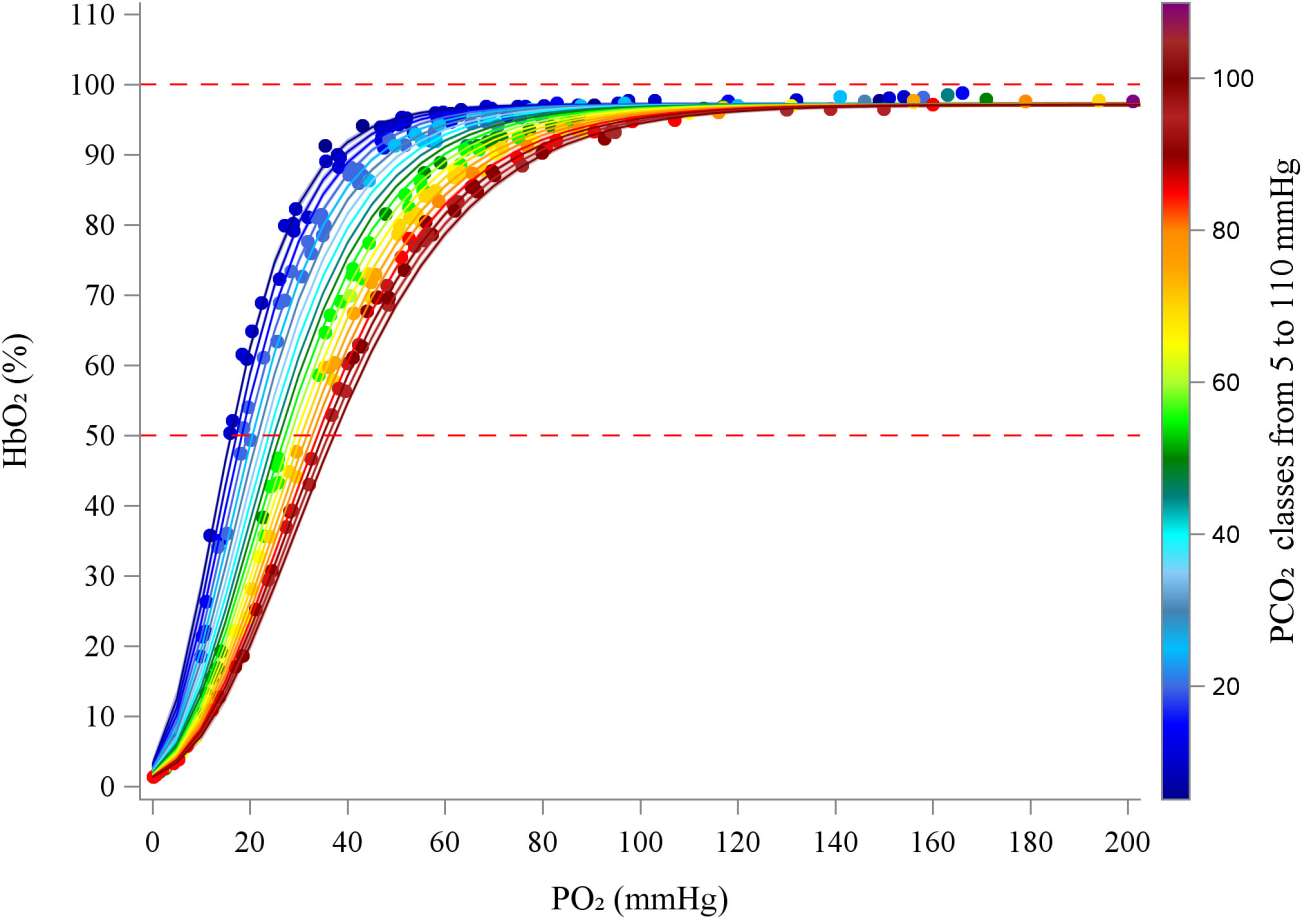
Figure shows oxygen–hemoglobin dissociation curves estimated using the Gompertz mixed model (lines) at PCO_2_ values ranging from 5 to 110 mmHg (in 5 mmHg increments), with experimental data points (dots) grouped into 5 mmHg intervals for visualization purposes. Experimental PCO_2_ values were categorized into groups, from 5 to 110 mmHg, centered on multiples of 5 mmHg, each spanning ± 2.5 mmHg (e.g., the group centered at 5 mmHg included values from 2.5 to 7.5 mmHg). Red dashed lines represent reference lines at 50 and 100% HbO_2_.

Figure 1 illustrates the oxygen–hemoglobin dissociation curves estimated using the Gompertz mixed model at PCO_2_ values ranging between 5 and 110 mmHg (in 5 mmHg increments), with experimental data points grouped into 5 mmHg intervals for visualization purposes. Experimental PCO_2_ values were categorized into groups, from 5 to 110 mmHg, centered on multiples of 5 mmHg, each spanning ± 2.5 mmHg (e.g., the group centered at 5 mmHg included values from 2.5 to 7.5 mmHg).

The values of P50 estimated by the Gompertz model at different PCO_2_ values (5–100 mmHg) are reported in Supplementary Table 16. The increase in PCO_2_ significantly shifted (*P* < 0.0001) the P50 values to the right following the equation *P*50 = 14.91 + 0.22⋅*PCO*_2_.

### 3.2 Electrolytes

Collinearity diagnostics showed no significant collinearity among the independent variables.

A detailed description of model selection procedures—including LRT results, AIC values, residual inspection, and graphical representations— and comparisons between experimental data points with the estimated relationships are provided in Supplementary material.

#### 3.2.1 Strong ion difference

**FIGURE 2:**
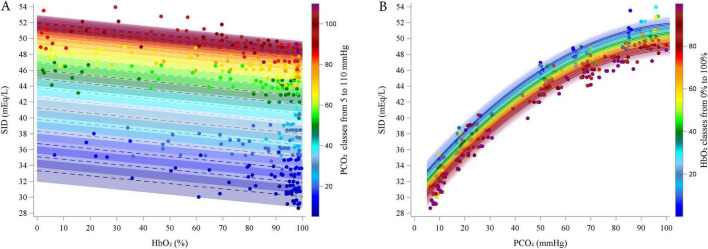
Estimated relationship (lines) between estimated SID and HbO_2_ at different PCO_2_ (5–110 by 5 mmHg, **A)** and between SID and PCO_2_ at different HbO_2_ (0–100% by 5% **B)**. Dots represent experimental points. categorized into groups, from 5 to 110 mmHg, centered on multiples of 5 mmHg, each spanning ± 2.5 mmHg (e.g., the group centered at 5 mmHg included values from 2.5 to 7.5 mmHg).

Preliminary graphical analyses suggested a linear relationship between SID and HbO_2_ and a quadratic relationship between SID and PCO_2_ (Supplementary Figures 10–13). The final polynomial multilevel model included HbO_2_ and PCO_2_ as predictors of both the intercept and the linear terms, as well as a quadratic term for PCO_2_. Random intercepts at the subject level and slopes for PCO_2_ were included. The model used an unstructured covariance matrix (AIC 892.8). Table 3 reports the estimated fixed effects parameters. Residual plots showed no autocorrelation, and bootstrap analysis (1,000 resamples) confirmed parameter reliability (see Supplementary material for details). Figure 2 illustrates the relationships between SID and HbO_2_ at different PCO_2_ levels (panel A; experimental data points grouped by 5 mmHg) and between SID and PCO_2_ and different HbO_2_ (panel B; data grouped in 5% intervals).

**TABLE 3:** Estimated parameters from the linear mixed model applied to describe the relationship between SID, HbO_2_, and PCO_2_.

Parameters	Estimate	Standard error	95% CIs		*P*-value
Intercept	31.5013	0.5911	30.0517	32.9509	<0.0001
HbO_2_: linear	−0.0327	0.0015	−0.0357	−0.0297	<0.0001
PCO_2_: linear	0.3763	0.0085	0.3584	0.3943	<0.0001
PCO_2_: quadratic	−0.0017	0.0001	−0.0018	−0.0016	<0.0001

CIs, confidence limits.

According to the model, SID decreased linearly with increasing HbO_2_ (coefficient = -0.0327, *P* < 0.0001). Conversely, SID initially increased with rising PCO_2_ (linear coefficient = 0.3763, *P* < 0.0001), but this effect diminished at higher PCO_2_ values, as indicated by the negative quadratic coefficient (coefficient = -0.0017, *P* < 0.0001). The interaction term was excluded from the final model due to lack of statistical significance.

#### 3.2.2 Chloride

Preliminary graphical analyses suggested a linear relationship between Cl^–^ and HbO_2_, and a quadratic relationship between Cl^–^ and PCO_2_ (see Supplementary Figures 21–24).

The final polynomial multilevel model included HbO_2_ and PCO_2_ as predictors of both the intercept and the linear terms, along with a quadratic term for PCO_2_. Random intercepts at the subject level and random slopes for PCO_2_ were included. The model was implemented using an unstructured covariance matrix (AIC = 846.0). The estimated fixed effects parameters are reported in Table 4.

**TABLE 4:** Estimated parameters from the linear mixed model applied to describe the relationship between chloride, HbO_2_ and PCO_2_.

Parameters	Estimate	Standard error	95% CI		*P*-value
Intercept	111.0100	0.5846	109.5700	112.4500	<0.0001
HbO_2_: linear	0.0242	0.0014	0.0214	0.0271	<0.0001
PCO_2_: linear	−0.2438	0.0068	−0.2575	−0.2302	<0.0001
PCO_2_: quadratic	0.0012	0.0001	0.0011	0.0014	<0.0001

CIs, confidence limits.

Residual plots showed no autocorrelation, and bootstrap analysis (1,000 resamples) confirmed parameter reliability (see Supplementary material for details).

**FIGURE 3:**
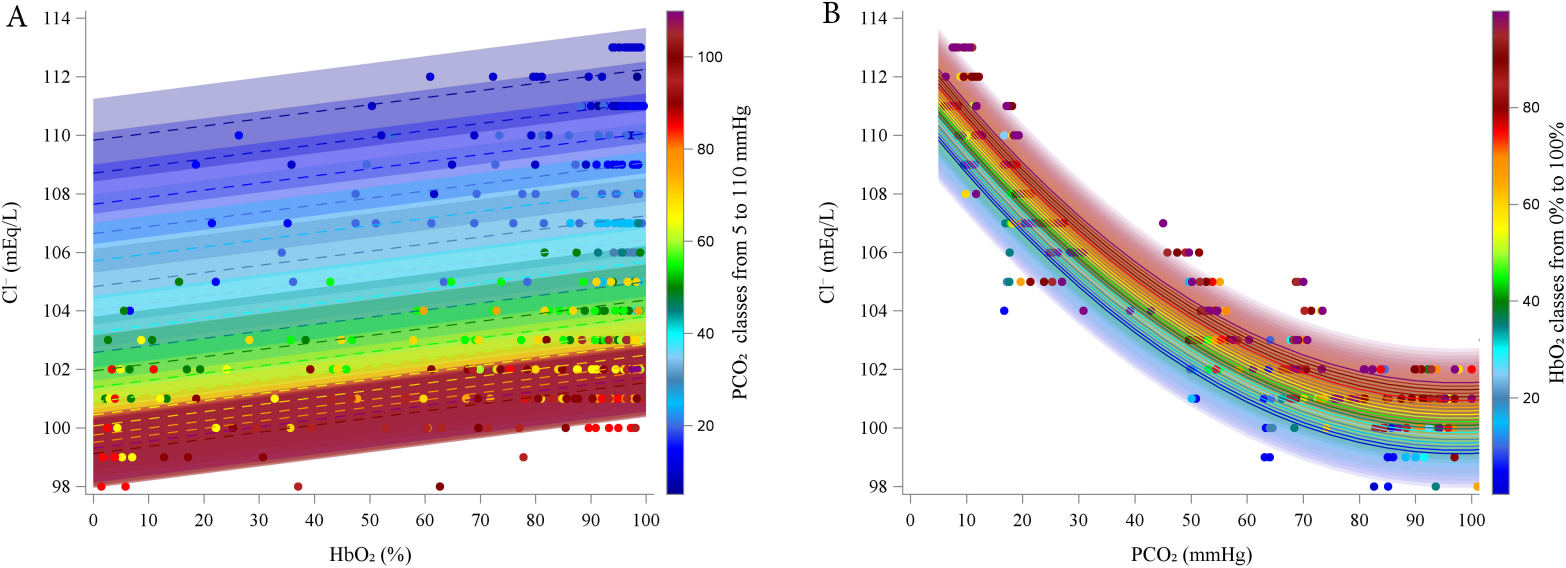
Estimated relationship (lines) between estimated Chloride and HbO_2_ at different PCO_2_ (5–110 by 5 mmHg, **A**) and between Chloride and PCO_2_ at different HbO_2_ (0–100% by 5%, **B**). Dots represent experimental points. categorized into groups, from 5 to 110 mmHg, centered on multiples of 5 mmHg, each spanning ± 2.5 mmHg (e.g., the group centered at 5 mmHg included values from 2.5 to 7.5 mmHg).

Figure 3 illustrates the relationships between Cl^–^ and HbO_2_ at different PCO_2_ levels (panel A; experimental data grouped in 5 mmHg intervals), and between Cl^–^ and PCO_2_ at different HbO_2_ levels (panel B; data grouped in 5% intervals).

According to the model, Cl^–^ increased linearly with increasing HbO_2_ (coefficient = 0.0242, *P* < 0.0001). In contrast, Cl^–^ decreased with rising PCO_2_ (linear coefficient = -0.2438, *P* < 0.0001), but this effect diminished at higher PCO_2_ levels, as indicated by the positive quadratic coefficient (quadratic coefficient = 0.0012, *P* < 0.0001). The interaction term was excluded from the final model due to lack of statistical significance.

#### 3.2.3 Sodium

**FIGURE 4:**
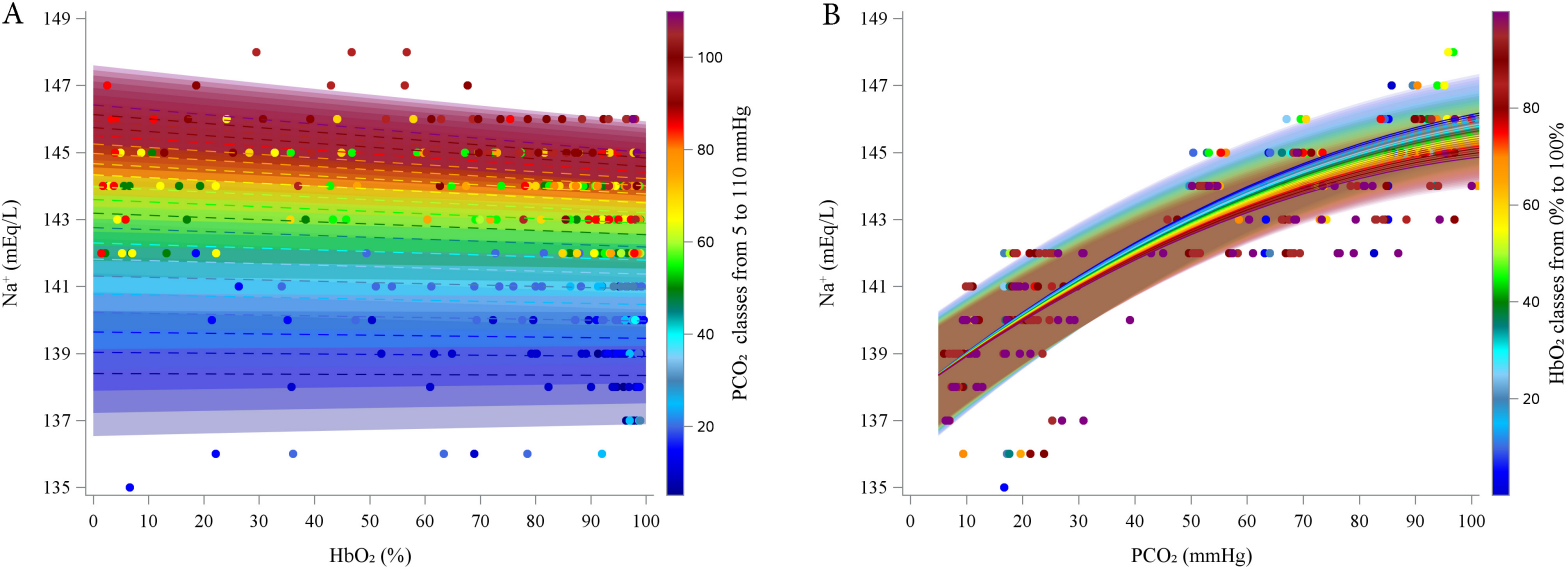
Estimated relationship (lines) between estimated Sodium and HbO_2_ at different PCO_2_ (5–110 by 5 mmHg, **A**) and between Sodium and PCO_2_ at different HbO2 (0–100% by 5%, **B**). Dots represent experimental points. categorized into groups, from 5 to 110 mmHg, centered on multiples of 5 mmHg, each spanning ± 2.5 mmHg (e.g., the group centered at 5 mmHg included values from 2.5 to 7.5 mmHg).

Preliminary graphical analyses suggested a linear relationship between Na^+^ and HbO_2_ and suggested a quadratic association between Na^+^ and PCO_2_ (Supplementary Figures 32–35). The final polynomial multilevel model included HbO_2_ and PCO_2_ as predictors of both the intercept and the linear terms, along with a quadratic term for PCO_2_. An interaction term between the HbO_2_ and PCO_2_ was also included. Random intercepts at the subject level and random slopes for HbO_2_ and PCO_2_ were included. The model was fitted using an unstructured covariance matrix (AIC 940.9). Table 5 reports the estimated fixed effects parameters of the model. Residual plots showed no autocorrelation, and bootstrap analysis (730 resamples) confirmed parameter reliability (see Supplementary material for details). Figure 4 represents the relationships between Na^+^ and HbO_2_ at different PCO_2_ (panel A; experimental data grouped in 5 mmHg intervals) and between Na^+^ and PCO_2_ and different HbO_2_ (panel B; data grouped in 5% intervals). As PCO_2_ increased, Na^+^ initially increased due to the positive linear coefficient (coefficient = 0.134, *P* < 0.0001), but this effect diminished at higher levels of PCO_2_, as shown by the negative quadratic coefficient (coefficient = -0.00050, *P* < 0.0001). The linear effect HbO_2_ was not statistically significant. However, the interaction between HbO_2_ and PCO_2_ was significant (coefficient = -0.0001, *P* = 0.030), suggesting that HbO_2_ slightly reduced Na^+^ levels, particularly at higher PCO_2_ levels. This interaction may reflect a subtle but significant modulation of Na^+^ by oxygenation status under varying CO_2_ conditions.

**TABLE 5:** Estimated parameters from the linear mixed model applied to describe the relationship between sodium, HbO_2_ and PCO_2_.

Parameters	Estimate	Standard error	95% CI		*P*-value
Intercept	137.7400	0.8170	135.8100	139.6700	<0.0001
HbO_2_: linear*	0.00007	0.0039	−0.0078	0.0079	0.986
PCO_2_: linear	0.1339	0.0100	0.1138	0.1540	<0.0001
PCO_2_: quadratic	−0.00050	0.000059	−0.00062	−0.00038	<0.0001
HbO_2_ and PCO_2_ interaction	−0.000130	0.000060	−0.000250	−0.000010	0.030

CIs, confidence limits.

*Not statistically significant.

#### 3.2.4 Correlations between electrolytes

**FIGURE 5:**
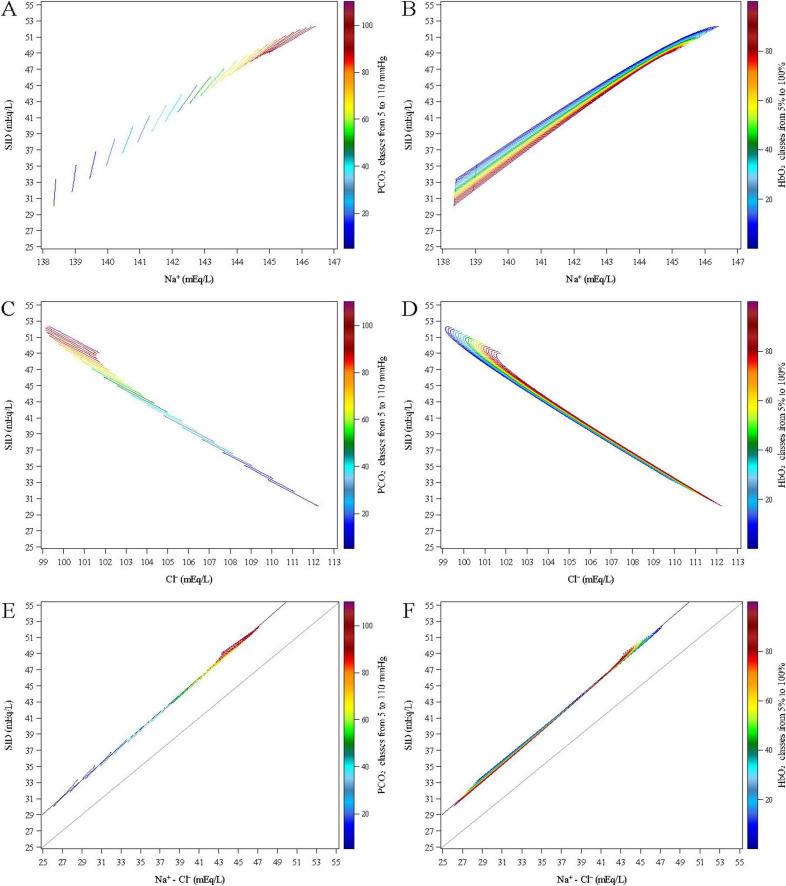
Estimated relationships between the SID and Na^+^ (upper panels **A,B**), Cl^–^ (middle panels **C,D**) and their difference (Na^+^-Cl^–^, lower panels **E,F**) across a simulated range of PCO_2_ (from 5 to 110 mmHg in 5 mmHg increments, left panels **A,C,E**) and of HbO_2_ (from 5 to 100% in 5% increments, right panels **B,D,F**).

Figure 5 shows the relationship between the SID and Na^+^, Cl^–^ and their difference (Na^+^-Cl^–^) across a simulated range of PCO_2_ (from 5 to 110 mmHg in 5 mmHg increments) and of HbO_2_ (from 5 to 100% in 5% increments). The values of SID, Na^+^, and Cl^–^ were computed using the equations derived from the previously described mixed linear models.

Figure 5A shows a direct relationship between Na^+^ and SID across varying levels PCO_2_. This relationship is modulated by HbO_2_ (Figure 5B), which drove the variation within each PCO_2_ value. SID consistently decreased with increasing HbO_2_, regardless of PCO_2_ level. At low PCO_2_, Na^+^ remained relatively stable across varying levels of HbO_2_ levels, resulting in a nearly linear relationship with a steep slope (e.g., slope = 56.67 at PCO_2_ = 5 mmHg). As PCO_2_ increased, the slope decreased markedly, approaching values close to 2 (e.g., 2.81, 2.66, and 2.53 at PCO_2_ = 90, 95, and 100 mmHg, respectively. At high PCO_2_ levels, the SID–Na^+^ curves tended to overlap, reflecting quadratic dependence on PCO_2_ in the underlying model equations.

Figures 5C,D show a negative relationship between SID and Cl^–^ across all PCO_2_ levels. As Cl^–^ increased, SID decreased, reflecting the inverse contribution of chloride to the strong ion balance. The relationship remained consistently linear across the full range of HbO_2_, with a constant slope of approximately -1.35. At higher PCO_2_ levels, the SID–Cl^–^ curves also tended to overlap, again due to the quadratic terms in the underlying equations.

The lower panels (E and F) show a positive linear relationship between SID and the difference Na^+^- Cl^–^ across all PCO_2_ levels. As Na^+^- Cl^–^ increased, SID increased proportionally, reflecting the direct contribution of strong cations and anions to the strong ion balance. The relationship is described by the equation:


$$SID=1.0549\cdot \left(Na^{+}-Cl^{-}\right)+2.6909$$

With an *R*^2^ = 0.999 (*P* < 0.0001), indicating that the Na^+^- Cl^–^ difference explained nearly all of the variability in SID. The median ratio (Na^+^-Cl^–^)/SID was 89.2% (IQR: 88.5–89.6). The intercept of the regression model likely reflects the contribution of other strong ions—such as potassium, calcium, and lactate.

For each fixed PCO_2_ value, the relationship remained linear across the full range of HbO_2_. As PCO_2_ increased, the slope of SID vs. Na^+^—Cl^–^ relationship slightly decreased (e.g., 1.32 at PCO_2_ = 5 mmHg vs. 0.88 at PCO_2_ = 110 mmHg). This reduction in slope was smaller than the difference between the slope of Na^+^ and that of Cl^–^, indicating that Na^+^—Cl^–^ was not a simple linear combination of the two variables. At higher PCO_2_ levels, the SID vs. Na^+^- Cl^–^ curves corresponding to different HbO_2_ values tended to overlap, again due to the quadratic terms in the model equations.

#### 3.2.5 Model validation

**FIGURE 6:**
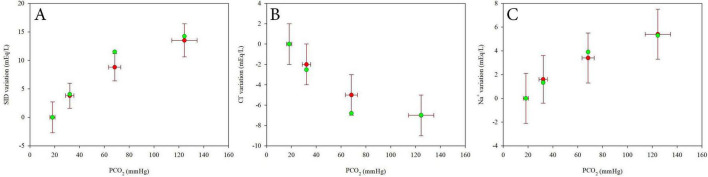
Comparison between experimental data from Langer et al. (9) (mean ± SD, red dots) and values estimated using linear mixed models (green dots) for SID variations **(A)**, Chloride variations **(B)** and Sodium variations **(C)** at varying PCO_2_. Values are expressed as difference from the first value at 2% CO_2_.

Supplementary Figure 43 and Figure 6 present the model validation using experimental data from 18 healthy subjects, as reported by Langer al. (11).

Values of SID, Na^+^ and Cl^–^ were estimated using the model at the average PCO_2_ values reported in the study and assuming HbO_2_ = 98%. Absolute values (Supplementary Figure 43) and the differences from the reference value at 5% CO_2_ (Figure 6) were plotted against PCO_2_ to assess model performance across a range of respiratory conditions. The model showed good agreement with the average experimental data, particularly in terms of the differences from the reference condition, supporting its validity and robustness.

## 4 Discussion

In this study, we investigated the hemoglobin-oxygen dissociation curves in whole human blood across a wide range of PO_2_ and PCO_2_. Using experimental data, we achieved a robust fit of the characteristic asymmetric sigmoidal shape of the dissociation curves at various levels of PCO_2_ by applying a Gompertz mixed-effects model. As expected, increasing PCO_2_ shifted the curves to the right, reflecting a decreased affinity of Hb for oxygen and thus promoting oxygen release. We also modeled the associated changes in plasma electrolyte concentrations using polynomial mixed-effects models. We observed that rising PCO_2_ and decreasing HbO_2_ were associated with an increased SID, primarily due to a reduction in chloride ions and a concurrent rise in sodium levels.

From an evolutionary perspective, the ability to utilize oxygen via mitochondrial oxidative phosphorylation—aerobic metabolism—provided a substantial advantage in energy production over anaerobic pathways. This metabolic efficiency supported the development of more complex life forms, including humans. On a broader scale, terrestrial life emerged through the interplay of two complementary biochemical cycles: photosynthesis and cellular respiration. Photosynthesis converts solar energy, water, and carbon dioxide into glucose and oxygen, while cellular respiration uses glucose and oxygen to generate carbon dioxide, water, and energy in the form of adenosine triphosphate (ATP). Interestingly, while oxygen is essential for aerobic life, it is also highly reactive and potentially toxic in the absence of effective antioxidant defenses. As a result, aerobic organisms have evolved to maintain relatively low oxygen reserves in the body, relying instead on continuous delivery to meet metabolic demands. In the average adult human, total body oxygen content is surprisingly limited, approximately 1.5 L, despite a high consumption rate of around 250 mL per minute. More than half of this oxygen is carried in the blood, predominantly bound to Hb. Only a small fraction is dissolved in plasma, yet this dissolved component determines the partial pressure of oxygen (PO_2_). For examples, at a PO_2_ of 100 mmHg, only about 0.31 mL of O_2_ is dissolved per 100 mL of blood, whereas over 19 mL of O_2_ is carried bound to Hb in a healthy individual.

The shape of the oxygen–hemoglobin dissociation curve provides several physiological advantages that optimize both oxygen uptake in the lungs and delivery to peripheral tissues. The upper plateau of the curve ensures near-complete Hb saturation even when alveolar PO_2_ is moderately reduced, thereby maintaining a favorable diffusion gradient from alveoli to blood. In contrast, the steep slope of the lower portion of the curve facilitates rapid oxygen unloading in peripheral tissues, where PO_2_ is lower.

Importantly, the position of the dissociation curve is not fixed but is dynamically modulated by several factors, including pH, PCO_2_, temperature, and 2,3-DPG. Among these, PCO_2_, central to our investigation, plays a particularly prominent role as originally described by Bohr. Elevated PCO_2_ levels, such as those found in metabolically active tissues, shift the curve to the right, decreasing Hb’s affinity for oxygen and promoting its release. This rightward shift helps maintain the necessary PO_2_ gradient that drives oxygen diffusion from Hb to tissues, ultimately enabling oxygen to reach the mitochondria where it is consumed.

Based on our data, assuming a venous PO_2_ of 40 mmHg, a PCO_2_ of 46 mmHg, and 15 g/dL of Hb, the resulting Hb saturation would be approximately 77.02%, corresponding to an oxygen content of 16.18 mL O_2_ per 100 mL of blood. Without the rightward shift caused by the increase in PCO_2_ from 40 mmHg (arterial) to 46 mmHg (venous), the same PO_2_ of 40 mmHg would result in a saturation of 79.53%, with an oxygen content of 16.71 mL/100 mL. We can therefore estimate that the Bohr effect, due solely to the 6 mmHg arterio-venous increase in PCO_2_, contributes to the release of more than 26 mL of O_2_ per minute, approximately 10% of total oxygen consumption, at constant PO_2_.

Compared to the pivotal findings originally reported by Bohr (3), we observed a smaller shift of the oxygen–hemoglobin dissociation curve in response to varying PCO levels (see Supplementary Figure 9). This difference may be partially explained by the use of animal blood in Bohr’s experiments. It is well established that Hb’s affinity for oxygen varies across species (12–14). While maintaining a similar quaternary structure, differences in primary amino acid sequences can significantly alter ligand-binding properties. These changes at specific protein sites affect Hb’s behavior in the presence of gases. Furthermore, differences exist in the types of organic phosphate molecules bound by Hb, for example, inositol-5-phosphate in birds, 2,3-DPG in mammals, and ATP and GTP in fish. These differences may confer an evolutionary advantage under extreme conditions, such as those faced by organisms living at low temperatures, animals undergoing prolonged apnea, or organisms adapted to flight (15).

In our study, we also investigated the behavior of plasma electrolytes in whole blood from healthy human subjects in response to variations in PO_2_ and PCO_2_. The experimental design was structured to eliminate confounding factors such as electrolyte exchange between blood and interstitial compartments or organs, fluid infusion, and renal filtration.

Significant changes in chloride ion concentrations [Cl^–^] were observed with PCO_2_ variations. Specifically, [Cl^–^] decreased as at increasing PCO_2_ levels. In contrast, plasma [Na^+^] showed the opposite behavior.

Variations in HbO_2_ were also associated with significant changes in [Cl^–^], but not in [Na^+^]. Higher [Cl^–^] were observed at HbO_2_ near 100%, while lower [Cl^–^] concentrations were found at lower Hb saturation.

This chloride behavior is consistent with the classic “chloride shift” described over a century ago by Hamburger (16, 17). As PCO_2_ increases, bicarbonate ions accumulate within red blood cells due to the activity of carbonic anhydrase. Cl^–^ ions are exchanged with bicarbonate, moving from the extracellular/plasma compartment into the erythrocytes. The chloride shift through the red blood cells membrane, can be the result of the Cl^–^/HCO_3_^–^ antiport via Anion Exchanger 1 (also known as Band 3), the most abundant transport protein in the red blood cells membrane (18).

Moreover, the observed changes in [Cl^–^] with varying HbO_2_ may be attributed to the differing binding capacities of the two Hb conformational states. In the oxygenated R-state, Hb has significantly lower affinity for chloride ions, protons, and organic phosphates, resulting in their release into the erythrocytic cytoplasm. Previous studies have reported the release of approximately 1.8 Cl^–^ per Hb tetramer during the T-to-R transition at pH 7.4 (19).

Prange et al. (8) reported no differences in [Na^+^] in human whole blood and plasma across physiological ranges of PO_2_ and PCO_2_ designed to mimic the arteriovenous difference. This contrasts with the findings of Langer et al. (20), who observed significant variations in both [Cl^–^] and [Na^+^] under varying gas pressures across an extracorporeal membrane oxygenation system. Similarly, Krbec et al. (11, 21) demonstrated comparable changes in [Cl^–^] and [Na^+^] in whole human blood by experimentally varying PCO_2_ levels.

Our results confirm the changes of both [Cl^–^] and [Na^+^] in response to variations in PCO_2_. While the mechanisms driving [Cl^–^] shifts are well established, the behavior of [Na^+^] remains less clear and requires further investigation. One hypothesis is that an increased intracellular negative charge, particularly during deoxygenation and bicarbonate accumulation, may favor the passive influx of positively charged ions such as Na^+^. Changes in intracellular [Na^+^] may also correlate with solute-free water movement across the erythrocyte membrane, a response to alterations in intracellular charge (22–24). Alternatively, changes in blood pH due to variations in gas pressures might affect the structure and charge of plasma proteins, altering their capacity to bind sodium. Consequently, Na^+^ could transit from the free ion pool to a protein-bound form, thereby contributing to the so-called Strong Ion Reserve (25), a buffer system consisting of ions reversibly bound to plasma proteins. According to Stewart’s model, both PCO_2_ and SID are independent variables influencing pH. However, our data suggests a more complex interplay between PCO_2_, Hb conformation, and SID. Although Stewart’s model primarily addresses equilibrium in a single-compartment system, it acknowledges both passive (as in the case of Cl^–^) and active ion shifts in response to PCO_2_ changes. In our experiment, plasma ion concentrations varied in a manner that appears to counteract the changes induced by PCO_2_ variation, potentially buffering pH changes. This agrees with the findings of Langer et al. (20), Prange et al. (8), and Krbec et al. (21), who noted that, without SID variations in response to increased PCO_2_, pH would change far more drastically during the arteriovenous transition.

Our data provide valuable insights into the dynamics of blood electrolyte changes following substantial shifts in PO_2_ and PCO_2_, which can occur in both physiological and pathological conditions, such as acute changes in minute ventilation or during extracorporeal treatments.

For instance, during extracorporeal respiratory support, blood entering the oxygenator undergoes a marked transition from deoxygenated, hypercapnic conditions to oxygenated, hypocapnic states. A clearer understanding of the mechanisms by which electrolyte concentrations across the red blood cell membrane respond to variations in gas partial pressures may help clinicians optimize extracorporeal support settings.

## 5 Limitations

First, we used a limited set of nominal PCO_2_ values (10, 20, 50, 70, and 90 mmHg), which left a portion of the physiological PCO_2_ range underrepresented. To mitigate this issue, we treated PCO_2_ as a continuous variable in the modeling process, leveraging the broader distribution of measured PCO_2_ values obtained from blood gas analysis. This approach allowed the model to interpolate across intermediate PCO_2_ levels not explicitly included in the experimental design. Importantly, the HbO_2_ vs. PO_2_ dissociation curves were highly precise, and the consistency of the model across the full PO_2_ range suggests that the estimated curves at untested PCO_2_ levels are reliable and physiologically plausible.

Second, since Hb saturation was titrated at 37°C by progressively reducing PO_2_ at constant PCO_2_, the duration of gas equilibration led to increased lactate levels at lower saturation values. This likely introduced a metabolic component influencing pH at the lowest HbO_2_ levels. To minimize this confounding effect, we excluded from analysis 16 data points in which lactate increased by more than 2 mmol/L compared to baseline.

It is important to note that the models used to describe SID and electrolytes are dependent on the chosen functional form of the equations. Although model selection was guided by statistical criteria to identify the best-fitting formulations, the use of polynomial mixed-effects models may have influenced the behavior of the curves, particularly at the extremes of the PCO_2_ range. For instance, what might physiologically correspond to a plateau could be misrepresented by a downward trend due to the curvature imposed by a polynomial function. This highlights the need for cautious interpretation of model predictions, especially outside the range of experimental data.

To further support our findings, we applied our equations to data extracted from a previously published study by Langer al. (11). Using their reported values, values of SID, Na^+^ and Cl^–^ were estimated using our models at the average PCO_2_ values reported in the study and assuming HbO_2_ = 98%. Absolute values (Supplementary Figure 43) and differences from the reference value at 5% CO_2_ (Figure 6) show good consistency between our model predictions and the experimental data from that study, reinforcing the physiological plausibility of our approach.

Finally, our study was conducted exclusively on blood from healthy individuals. Thus, results may differ under pathological conditions.

## 6 Conclusion

Changes in PCO_2_ in human whole blood lead to a significant shift in the hemoglobin-oxygen dissociation curve (Bohr effect) and markedly alter plasma electrolyte concentrations. The rightward shift of the curve with increasing PCO_2_ can be effectively modeled using a Gompertz mixed-effects model. In contrast, the changes in plasma electrolyte concentrations, specifically, the reduction in chloride ions and the concurrent increase in sodium levels with rising PCO_2_, can be described using polynomial mixed-effects models. Understanding and modeling this physiology may help clarify not only the responses of healthy individuals exposed to extreme conditions but also, potentially, those of patients with underlying pathologies.

## Data Availability

The raw data supporting the conclusions of this article will be made available by the authors, without undue reservation.
