# Supplementary material for: In vitro characterization of hemoglobin oxygen dissociation curves and electrolyte shifts in human blood under varying PCO2
Source: Front Med (Lausanne). 2026 Jan 12;12:1708274. doi: 10.3389/fmed.2025.1708274 (PMC12833093; doi:10.3389/fmed.2025.1708274)
Supplement: Supplementary file 1 [file Data_Sheet_1.docx]

Supplementary Material

In Vitro Characterization of Hemoglobin Oxygen Dissociation Curves and Electrolyte Shifts in Human Blood under Varying PCO₂

Carlo Valsecchi, Eleonora Carlesso, Michele Battistin, Sebastiano M Colombo, Emanuele Cattaneo, Francesca Gori, Thomas Langer, Giacomo Grasselli, Alberto Zanella

# Additional Results

## A total of 361 samples were collected, 15 (4.16%) samples were excluded from statistical analyses due to the increase in lactate levels within the sample of more than 2 mEq/L compared to baseline.

| **Nominal PCO_2_ (mmHg)** | **N.** | **Median** | **Lower Quartile** | **Upper Quartile** | **Minimum** | **Maximum** |
| --- | --- | --- | --- | --- | --- | --- |
| **10** | **65** | 9.9 | 8.2 | 11.0 | 6.0 | 39.1 |
| **20** | **77** | 19.5 | 17.7 | 24.0 | 16.6 | 30.8 |
| **50** | **61** | 52.4 | 50.4 | 54.5 | 42.9 | 61.0 |
| **70** | **64** | 68.7 | 67.0 | 70.7 | 63.0 | 79.3 |
| **90** | **79** | 91.5 | 85.1 | 95.8 | 78.4 | 111.0 |

*Table S1: Measured PCO_2_ distribution according to nominal PCO_2_ values*

| **Nominal PCO_2_ (mmHg)** | **N.** | **Median** | **Lower Quartile** | **Upper Quartile** | **Minimum** | **Maximum** |
| --- | --- | --- | --- | --- | --- | --- |
| **10** | **65** | 61.1 | 37.9 | 151.0 | 11.7 | 696.0 |
| **20** | **77** | 56.9 | 31.8 | 98.1 | 4.6 | 721.0 |
| **50** | **61** | 55.4 | 25.8 | 85.8 | 1.5 | 674.0 |
| **70** | **64** | 57.5 | 32.4 | 99.9 | 2.5 | 664.0 |
| **90** | **79** | 56.4 | 32.5 | 94.0 | 0.1 | 656.0 |

*Table S2: Measured PO_2_ distribution according to nominal PCO_2_ values*

| **Nominal PCO_2_ (mmHg)** | **N.** | **Median** | **Lower Quartile** | **Upper Quartile** | **Minimum** | **Maximum** |
| --- | --- | --- | --- | --- | --- | --- |
| **10** | 65 | 96.0 | 89.6 | 98.0 | 35.8 | 99.2 |
| **20** | 77 | 92.9 | 76.0 | 97.3 | 6.6 | 99.6 |
| **50** | 61 | 86.2 | 46.8 | 94.8 | 2.1 | 98.9 |
| **70** | 64 | 84.6 | 52.8 | 95.3 | 2.90 | 98.7 |
| **90** | 79 | 78.8 | 46.7 | 93.3 | 1.4 | 98.7 |

*Table S3: Measured HbO_2_ distribution according to nominal PCO_2_ values*

## Oxygen–hemoglobin dissociation curve

The hemoglobin dissociation curve is characterized by a sigmoidal (S-shaped) form, reflecting the binding of oxygen to the hemoglobin subunits. In this paper we used the three-parameter Gompertz curve for modelling the curves which, due to its flexibility, is a good candidate in capturing sigmoidal shapes. We applied a mixed Gompertz model with proc NLMIXED. Models with random effects were also tested for appropriateness.

The equation used for the model was:

$${HbO}_{2}=a\cdot e^{{-e}^{\frac{-(PO_{2}-x0)}{b}}}$$

Where:

**a** represents the upper asymptote of the curve. It is the maximum value that f can reach as x approaches infinity.

**x0** represents the inflection point of the curve along the x x-axis. It is the value of x at which the growth rate is the highest.

**b** controls the growth rate and the steepness of the curve. It determines how quickly the function approaches its asymptote.

### Examining experimental data and individual curves fittings

Figure S1 shows Oxygen–hemoglobin dissociation curves (all subjects experimental data points) obtained after titrating the blood samples at different nominal PCO_2_ values.


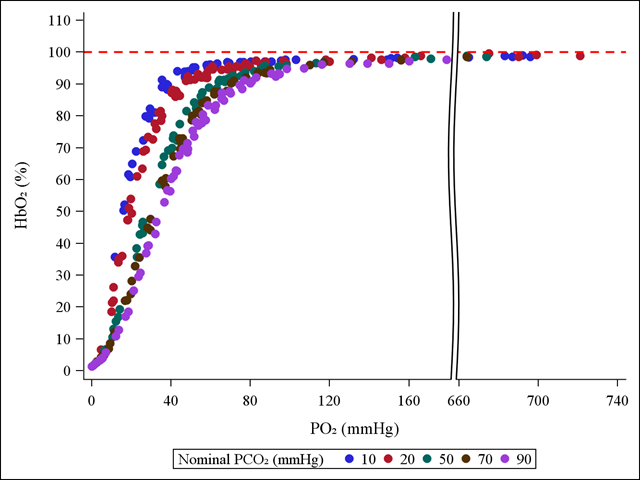


*Figure S1 - Oxygen–hemoglobin dissociation curves (all subjects experimental data points) obtained after titrating the blood samples at different nominal PCO_2_ values. Red dashed line represent reference at 100%. The x-axis was hidden from 180 to 660 mmHg.*

To evaluate the distribution of the parameters in our population and to assess which parameters should be included in the model (fixed and random effects) we fitted the individual subject data at different nominal PCO_2_ values with proc NLIN (initial guess a=100, b=10, x0=11).

*Figure S2 – Fitting with Gompertz curve of the individual subjects (sbj) experimental data at different nominal PCO_2_ values. Green dots represent experimental data points, the black lines represent the fitting curves, the red dashed lines represent the reference at 100%*

*A – nominal PCO_2_= 10 mmHg*


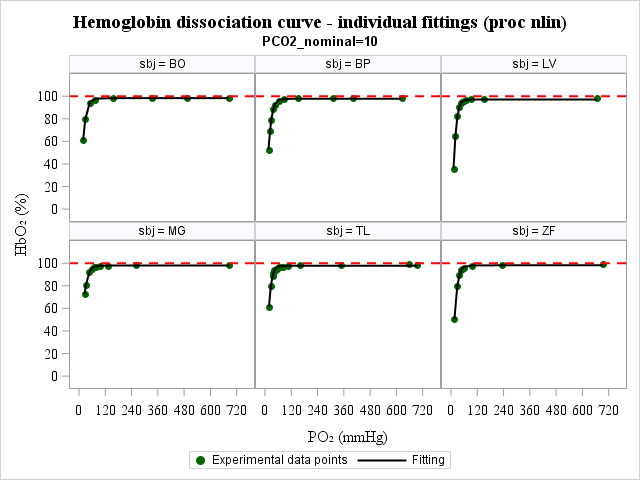


*B – nominal PCO_2_= 20 mmHg*


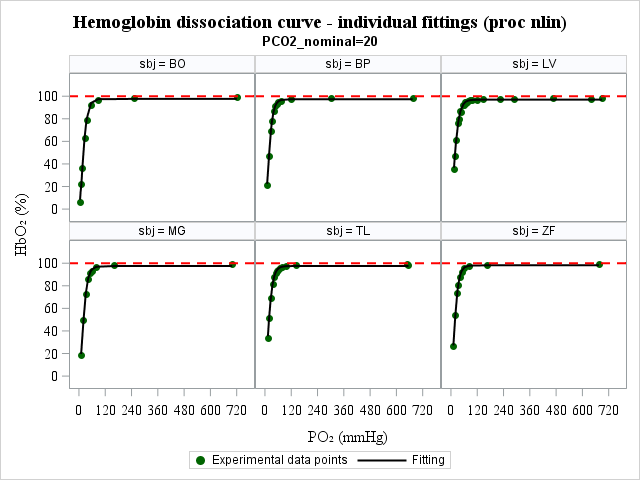


*C – nominal PCO_2_= 50 mmHg*


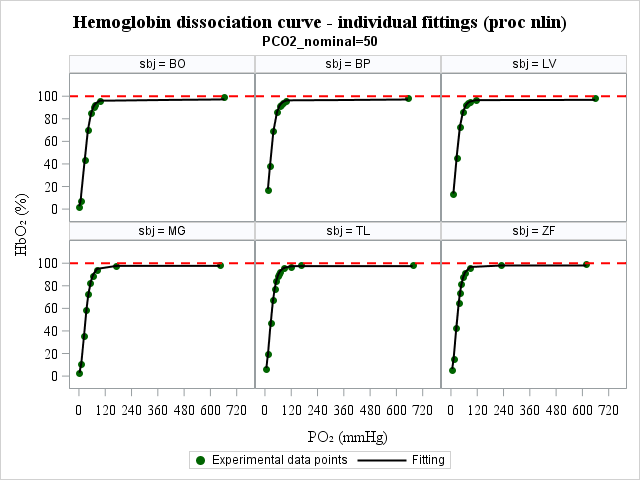


*D – nominal PCO_2_= 70 mmHg*


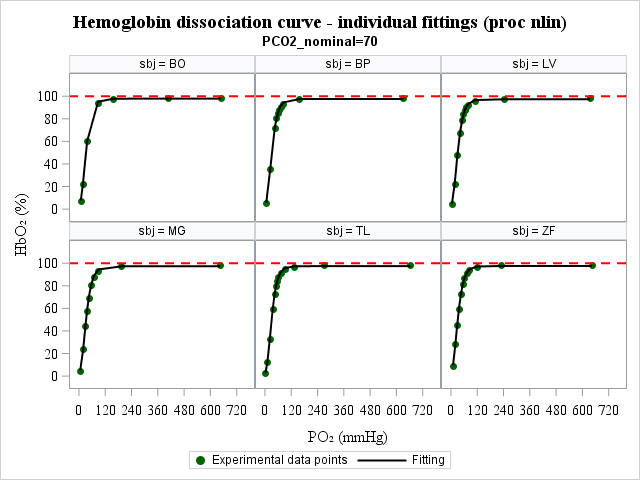


*E – nominal PCO_2_= 90 mmHg*


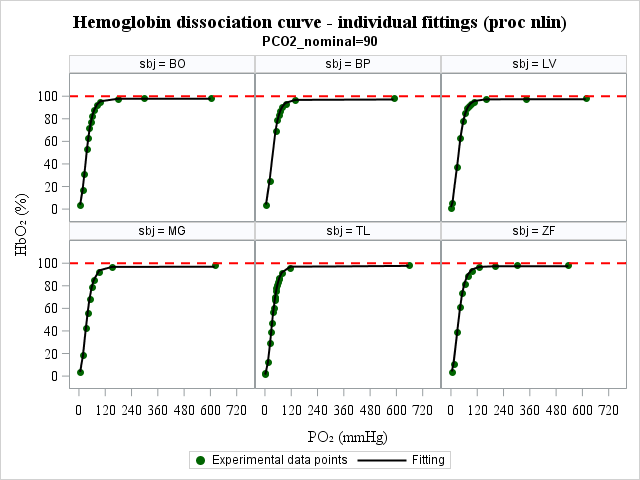


The estimated parameters, as well as estimated values and residuals, were obtained for each subject at each nominal PCO_2_.

The residual inspection of the residual (overall and of individual subjects) indicated that the model adequately described data.

*Figure S3 - Residuals distribution (left panel) and QQ plot (right panel). Parameters of the normal distribution: Mean -0.02247; SD 0.72843; Normality Test (Shapiro-Wilk) P=0.058.*

Bivariate distribution of the estimated parameters showed that **a** and **b** (correlation coefficient = -0.28034, p<.0001), as well as **a** and **x0** (correlation coefficient = -0.7918, p<.0001)**,** only moderately correlated, while **b** and **x0** strongly correlated (correlation coefficient = 0.95801, p<.0001). We did not perform a reparameterization as we still did not know the meaning of new parameters.


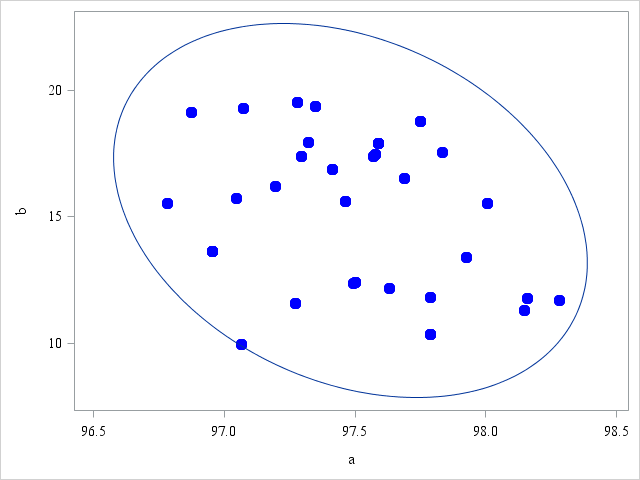

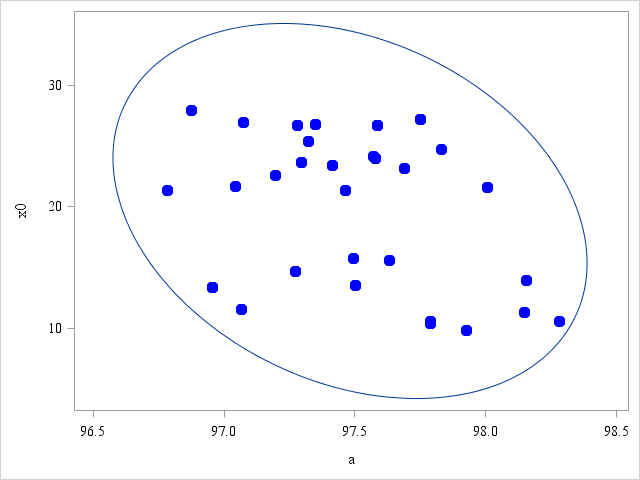

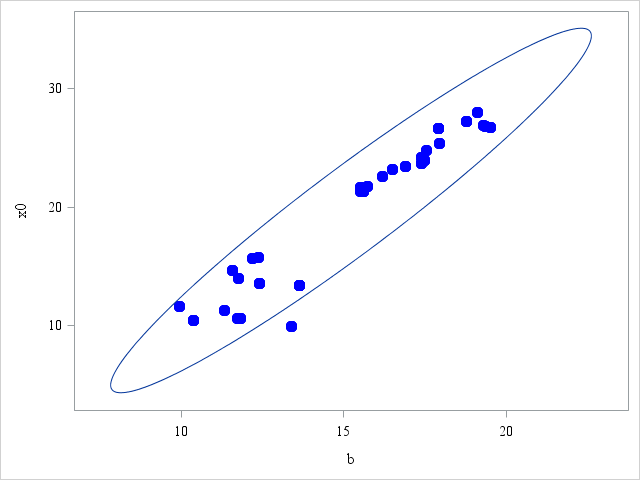


*Figure S4 - Bivariate distribution of the model parameters and 95% confidence ellipse*

We plotted the individual estimates and 95% confidence intervals (CIs) at different nominal PCO_2_ values to investigate if the parameters are fixed or random. Graphs showed that CIs (especially **b** and **x0**) did not overlap for different subjects in some conditions. This suggests that parameters should be considered as random and fixed (random)

*Figure S5 – Individual estimates and 95% confidence intervals at different nominal PCO_2_ values of the individual estimated parameters*

*Parameter = a*


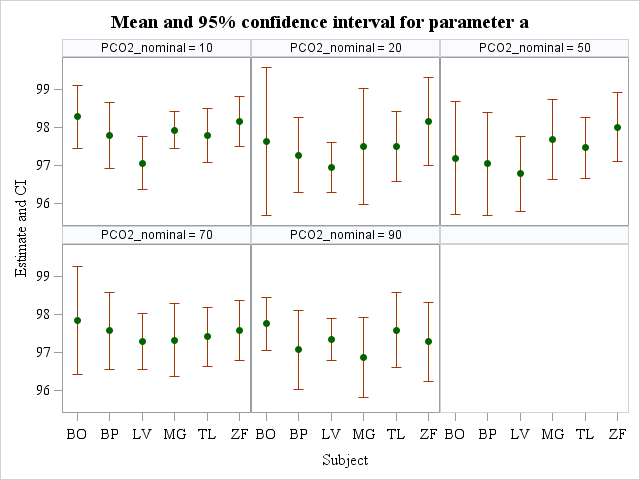


*Parameter = b*


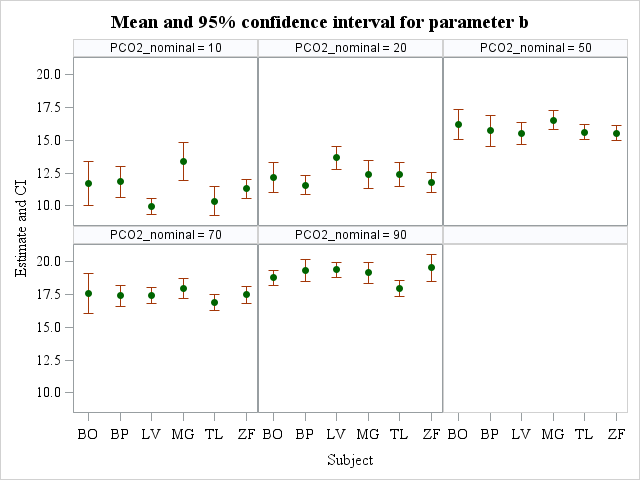


*Parameter = x0*


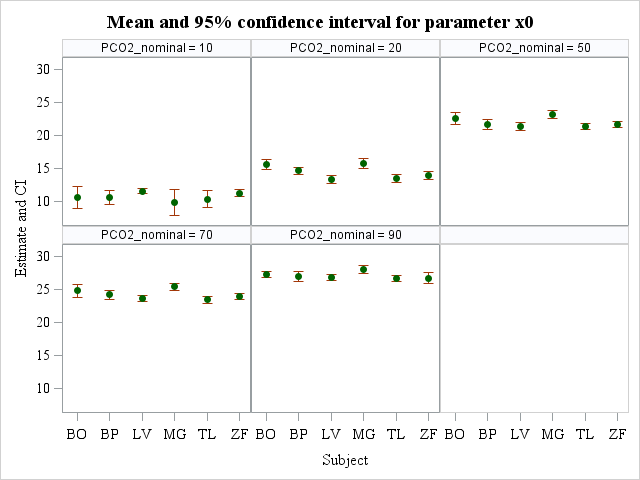


The mean ± standard deviation (SD) parameter estimates obtained by the individual fittings are reported in table S4:

| **Parameter** | **N. obs** | **Mean** | **SD** |
| --- | --- | --- | --- |
| **a** | **30** | 97.5043671 | 0.3890611 |
| **b** | **30** | 15.2059958 | 3.0347756 |
| **x0** | **30** | 19.6783884 | 6.2786502 |

*Table S4: mean ± SD parameter estimates obtained by the individual fittings*

The effect of nominal PCO_2_ on the parameters estimated is reported in the table S5. As expected, a (the upper asymptote) is not affected by PCO_2_, on the contrary b and x0 are right-shifted at increasing nominal PCO_2_ values.

| **Parameter** | **Nominal PCO_2_** | **N. sbj** | **Mean** | **SD** |
| --- | --- | --- | --- | --- |
| **a** | **10** | **6** | 97.8335726 | 0.4247153 |
|  | **20** | **6** | 97.5021692 | 0.3996108 |
|  | **50** | **6** | 97.3640537 | 0.4462774 |
|  | **70** | **6** | 97.5025623 | 0.2011258 |
|  | **90** | **6** | 97.3194778 | 0.3217815 |
| **b** | **10** | **6** | 11.4279943 | 1.2220165 |
|  | **20** | **6** | 12.3271477 | 0.7288019 |
|  | **50** | **6** | 15.8440713 | 0.4127187 |
|  | **70** | **6** | 17.4335296 | 0.3369814 |
|  | **90** | **6** | 18.9972362 | 0.5869413 |
| **x0** | **10** | **6** | 10.6979553 | 0.6276166 |
|  | **20** | **6** | 14.4590517 | 1.0520676 |
|  | **50** | **6** | 21.9540708 | 0.7555260 |
|  | **70** | **6** | 24.2275721 | 0.7412883 |
|  | **90** | **6** | 27.0532919 | 0.4900370 |

*Table S5: mean ± SD parameter estimates obtained by the individual fittings at different nominal PCO_2_ values*

### Building the Mixed Model

The model was initially built with no random effects and with all the parameters depending on PCO_2_ considered as a continuous variable.

a = a_fix_ + a_PCO2_ · PCO_2_;

b = b_fix_ + b_PCO2_ · PCO_2_;

x0 = x0_fix_ + x0_PCO2_ · PCO_2_;

The mean values of the parameters estimated by the individual fittings (table S4) were used as starting values for the fixed part of the parameters. The starting values for parameters depending on PCO_2_ were set at 1 for **b** and **x0** and a value close to 0 for **a**. The term **s2e** represents the residual variance of the model. This parameter captures the variability in the response variable HbO_2_ that is not explained by the model. The convergence criterion was set at 0.000000000000001 and the number of iterations at 1000000.

| **Parameter** | **Estimate** | **Standard Error** | **DF** | **P** | **95% Confidence Limits** | |
| --- | --- | --- | --- | --- | --- | --- |
| **a_fix_** | 97.0503 | 0.2635 | 346 | <.0001 | 96.5321 | 97.5686 |
| **a_PCO2_** | 0.002118 | 0.005048 | 346 | 0.6751 | -0.00781 | 0.01205 |
| **b_fix_** | 9.1881 | 0.2319 | 346 | <.0001 | 8.7319 | 9.6442 |
| **b_PCO2_** | 0.1073 | 0.004492 | 346 | <.0001 | 0.09846 | 0.1161 |
| **x0_fix_** | 11.1245 | 0.1663 | 346 | <.0001 | 10.7975 | 11.4516 |
| **x0_PCO2_** | 0.1796 | 0.003174 | 346 | <.0001 | 0.1733 | 0.1858 |
| **s2e** | 3.2706 | 0.2487 | 346 | <.0001 | 2.7816 | 3.7597 |

*Table S6: Estimated parameters for the nonlinear mixed model with no random effects and with all the parameters depending on PCO_2_. DF =degrees of freedom*

The AIC of the model was 1405.9. As expected, **a** did not depend on PCO_2_ (P=0.6751) consequently **a_PCO2_** was excluded from the model.

| **Parameter** | **Estimate** | **Standard Error** | **DF** | **P** | **95% Confidence Limits** | |
| --- | --- | --- | --- | --- | --- | --- |
| **a** | 97.1399 | 0.1548 | 346 | <.0001 | 96.8354 | 97.4444 |
| **b_fix_** | 9.2308 | 0.2089 | 346 | <.0001 | 8.8199 | 9.6418 |
| **b_PCO2_** | 0.1062 | 0.003742 | 346 | <.0001 | 0.09889 | 0.1136 |
| **x0_fix_** | 11.1305 | 0.1659 | 346 | <.0001 | 10.8043 | 11.4568 |
| **x0_PCO2_** | 0.1793 | 0.003120 | 346 | <.0001 | 0.1732 | 0.1855 |
| **s2e** | 3.2723 | 0.2488 | 346 | <.0001 | 2.7830 | 3.7616 |

*Table S7: Estimated parameters for the nonlinear mixed model with no random effects and with only b and x0 depending on PCO_2_. DF =degrees of freedom*

AIC = 1404.1

#### Adding random effects

We then performed a model adding one random effect **u1** on **a** and the effect of PCO_2_ on **b** and **x0.** The initial guess for s2u1 (variance of the random effect **u1**) was set at 0.01.

| **Parameter** | **Estimate** | **Standard Error** | **DF** | **P** | **95% Confidence Limits** | |
| --- | --- | --- | --- | --- | --- | --- |
| **a** | 97.1254 | 0.2906 | 5 | <.0001 | 96.3783 | 97.8724 |
| **b_fix_** | 9.2324 | 0.2015 | 5 | <.0001 | 8.7145 | 9.7502 |
| **b_PCO2_** | 0.1066 | 0.003603 | 5 | <.0001 | 0.09733 | 0.1158 |
| **x0_fix_** | 11.1261 | 0.1597 | 5 | <.0001 | 10.7157 | 11.5366 |
| **x0_PCO2_** | 0.1795 | 0.003005 | 5 | <.0001 | 0.1718 | 0.1872 |
| **s2e** | 3.0249 | 0.2320 | 5 | <.0001 | 2.4285 | 3.6214 |
| **s2u1** | 0.3706 | 0.2610 | 5 | 0.2150 | -0.3005 | 1.0416 |

*Table S8: Estimated parameters for the nonlinear mixed model with one random effect on a and with only b and x0 depending on PCO_2_. DF =degrees of freedom*

As expected, the random effect on **a** (the upper asymptote of the curve) was not significant (p = 0.2150). AIC = 1389.5.

The next step was performed with one random effect **u2** on **b** and the effect of PCO_2_ on **b** and **x0.** The initial guess for s2u2 (variance of the random effect **u2**) was 0.01. “Trureg” option was added otherwise the gradient of the objective function cannot be computed during the optimization process.

| **Parameter** | **Estimate** | **Standard Error** | **DF** | **P** | **95% Confidence Limits** | |
| --- | --- | --- | --- | --- | --- | --- |
| **a** | 97.1345 | 0.1466 | 5 | <.0001 | 96.7577 | 97.5113 |
| **b_fix_** | 9.2461 | 0.3128 | 5 | <.0001 | 8.4419 | 10.0503 |
| **b_PCO2_** | 0.1072 | 0.003547 | 5 | <.0001 | 0.09806 | 0.1163 |
| **x0_fix_** | 11.1365 | 0.1566 | 5 | <.0001 | 10.7340 | 11.5390 |
| **x0_PCO2_** | 0.1791 | 0.002947 | 5 | <.0001 | 0.1715 | 0.1867 |
| **s2e** | 2.9161 | 0.2237 | 5 | <.0001 | 2.3412 | 3.4911 |
| **s2u2** | 0.3512 | 0.2333 | 5 | 0.1926 | -0.2486 | 0.9510 |

*Table S9: Estimated parameters for the nonlinear mixed model with one random effect on b and with only b and x0 depending on PCO_2_. DF =degrees of freedom*

The random effect was not significant (p = 0.1926). AIC = 1378.7.

The last model with a single random effect was performed adding one random effect **u3** on **x0** and the effect of PCO_2_ on **b** and **x0.** The initial guess for s2u3 (variance of the random effect **u3**) was 0.01. Trureg option was added.

| **Parameter** | **Estimate** | **Standard Error** | **DF** | **P** | **95% Confidence Limits** | |
| --- | --- | --- | --- | --- | --- | --- |
| **a** | 97.1446 | 0.1359 | 5 | <.0001 | 96.7953 | 97.4940 |
| **b_fix_** | 9.2438 | 0.1859 | 5 | <.0001 | 8.7658 | 9.7217 |
| **b_PCO2_** | 0.1064 | 0.003310 | 5 | <.0001 | 0.09791 | 0.1149 |
| **x0_fix_** | 11.2255 | 0.3417 | 5 | <.0001 | 10.3470 | 12.1039 |
| **x0_PCO2_** | 0.1788 | 0.002813 | 5 | <.0001 | 0.1716 | 0.1861 |
| **s2e** | 2.5228 | 0.1935 | 5 | <.0001 | 2.0254 | 3.0203 |
| **s2u3** | 0.5667 | 0.3465 | 5 | 0.1629 | -0.3240 | 1.4574 |

*Table S10: Estimated parameters for the nonlinear mixed model with one random effect on x0 and with only b and x0 depending on PCO_2_. DF =degrees of freedom*

The random effect was not significant (p = 0.1629). AIC = 1333.8.

We then tested 3 models combining 2 random effects on the 3 parameters.

Model with 2 random effects (**u1** and **u2**) on **a** and **b** and effect of PCO_2_ on **b** and **x0**. The parameters **g11**, **g12** and **g22** represent the variances and covariance of the random parameters (initial guesses: g11=0.23, g21=-2.2378, g22=500). Trustreg e First-Order Integrated Random Effects were applied.

| **Parameter** | **Estimate** | **Standard Error** | **DF** | **P** | **95% Confidence Limits** | |
| --- | --- | --- | --- | --- | --- | --- |
| **a** | 97.0510 | 0.1578 | 4 | <.0001 | 96.6129 | 97.4890 |
| **b_fix_** | 9.2185 | 0.2441 | 4 | <.0001 | 8.5409 | 9.8961 |
| **b_PCO2_** | 0.1072 | 0.003521 | 4 | <.0001 | 0.09742 | 0.1170 |
| **x0_fix_** | 11.1147 | 0.1575 | 4 | <.0001 | 10.6775 | 11.5519 |
| **x0_PCO2_** | 0.1792 | 0.002958 | 4 | <.0001 | 0.1709 | 0.1874 |
| **s2e** | 2.9287 | 0.2269 | 4 | 0.0002 | 2.2988 | 3.5585 |
| **g11** | 0.02264 | 0.07687 | 4 | 0.7830 | -0.1908 | 0.2361 |
| **g21** | -0.1885 | 0.08791 | 4 | 0.0986 | -0.4325 | 0.05559 |
| **g22** | 0.1389 | 0.1222 | 4 | 0.3191 | -0.2003 | 0.4780 |

*Table S11: Estimated parameters for the nonlinear mixed model with two random effects on a and b and with only b and x0 depending on PCO_2_. DF =degrees of freedom*

The estimates for the variances and covariance of the random effects (g11, g21, g22) are not statistically significant, as indicated by their high p-values. This suggests that the random effects may not be contributing significantly to the model. AIC = 1376.3

Model with 2 random effects (**u1** and **u3**) on **a** and **x0** and effect of PCO_2_ on **b** and **x0**. The parameters **g11**, **g13** and **g33** represent the variances and covariance of the random parameters (initial guesses: g11=0.2, g21=0, g22=2).

| **Parameter** | **Estimate** | **Standard Error** | **DF** | **P** | **95% Confidence Limits** | |
| --- | --- | --- | --- | --- | --- | --- |
| **a** | 97.1720 | 0.1923 | 4 | <.0001 | 96.6382 | 97.7058 |
| **b_fix_** | 9.2379 | 0.1836 | 4 | <.0001 | 8.7281 | 9.7476 |
| **b_PCO2_** | 0.1067 | 0.003271 | 4 | <.0001 | 0.09764 | 0.1158 |
| **x0_fix_** | 11.2312 | 0.3332 | 4 | <.0001 | 10.3061 | 12.1562 |
| **x0_PCO2_** | 0.1788 | 0.002776 | 4 | <.0001 | 0.1711 | 0.1865 |
| **s2e** | 2.4591 | 0.1902 | 4 | 0.0002 | 1.9310 | 2.9872 |
| **g11** | 0.1093 | 0.1058 | 4 | 0.3598 | -0.1845 | 0.4032 |
| **g31** | -0.02892 | 0.1347 | 4 | 0.8405 | -0.4029 | 0.3451 |
| **g33** | 0.5352 | 0.3326 | 4 | 0.1829 | -0.3883 | 1.4588 |

*Table S12: Estimated parameters for the nonlinear mixed model with two random effects on a and x0 and with only b and x0 depending on PCO_2_. DF =degrees of freedom*

The estimates for the variances and covariance of the random effects (g11, g31, g33) are not statistically significant, as indicated by their high p-values, consequently these random effects may not be contributing significantly to the model. AIC = 1334.2

Model with 2 random effects (**u2** and **u3**) on **b** and **x0** and effect of PCO_2_ on **b** and **x0**. The parameters **g22**, **g32** and **g33** represent the variances and covariance of the random parameters (initial guesses: g22=0.2, g32=0, g33=2).

| **Parameter** | **Estimate** | **Standard Error** | **DF** | **P** | **95% Confidence Limits** | |
| --- | --- | --- | --- | --- | --- | --- |
| **a** | 97.1506 | 0.1345 | 4 | <.0001 | 96.7772 | 97.5241 |
| **b_fix_** | 9.2363 | 0.2235 | 4 | <.0001 | 8.6156 | 9.8569 |
| **b_PCO2_** | 0.1070 | 0.003272 | 4 | <.0001 | 0.09794 | 0.1161 |
| **x0_fix_** | 11.2285 | 0.3145 | 4 | <.0001 | 10.3553 | 12.1017 |
| **x0_PCO2_** | 0.1786 | 0.002769 | 4 | <.0001 | 0.1709 | 0.1863 |
| **s2e** | 2.4454 | 0.1893 | 4 | 0.0002 | 1.9199 | 2.9709 |
| **g22** | 0.09817 | 0.08898 | 4 | 0.3318 | -0.1489 | 0.3452 |
| **g32** | 0.1005 | 0.1177 | 4 | 0.4412 | -0.2263 | 0.4274 |
| **g33** | 0.4636 | 0.2918 | 4 | 0.1873 | -0.3466 | 1.2739 |

*Table S13: Estimated parameters for the nonlinear mixed model with two random effects on b and x0 and with only b and x0 depending on PCO_2_. DF =degrees of freedom*

Similarly, to the previous models, the estimates for the variances and covariance of the random effects (g22, g32, g33) are not statistically significant, as indicated by their high p-values. Consequently, this suggests that the random effects may not be contributing significantly to the model. AIC = 1331.9

Finally, we tested a model with 3 random effects (**u1**, **u2** and **u3**) on **a**, **b** and **x0** and the effect of PCO_2_ on **b** and **x0.** The parameters **g11, g21, g22, g31, g32, g33**represent the variances and covariance of the random parameters (initial guesses: g11=0.09028, g21=-0.2061, g22=0.03395, g31=-0.01383, g32=0.1616, g33=2.3191).

| **Parameter** | **Estimate** | **Standard Error** | **DF** | **P** | **95% Confidence Limits** | |
| --- | --- | --- | --- | --- | --- | --- |
| **a** | 97.6377 | . | 3 | . | . | . |
| **b_fix_** | 9.4335 | 0.3983 | 3 | 0.0002 | 8.1659 | 10.7011 |
| **b_PCO2_** | 0.09988 | 0.003150 | 3 | <.0001 | 0.08986 | 0.1099 |
| **x0_fix_** | 16.9669 | 0.5500 | 3 | <.0001 | 15.2165 | 18.7174 |
| **x0_PCO2_** | 0.1846 | 0.002574 | 3 | <.0001 | 0.1764 | 0.1928 |
| **s2e** | 2.0398 | 0.1581 | 3 | 0.0010 | 1.5365 | 2.5431 |
| **g11** | 2.9659 | 2.5587 | 3 | 0.3303 | -5.1769 | 11.1087 |
| **g31** | -0.6954 | . | 3 | . | . | . |
| **g33** | 11.2016 | 2.8040 | 3 | 0.0281 | 2.2781 | 20.1252 |
| **g21** | 1.5430 | . | 3 | . | . | . |
| **g22** | 1.4490 | 1.0269 | 3 | 0.2531 | -1.8191 | 4.7171 |
| **g32** | -2.9694 | 0.9068 | 3 | 0.0466 | -5.8552 | -0.08354 |

*Table S14: Estimated parameters for the nonlinear mixed model with three random effects on a, b and x0 and with only b and x0 depending on PCO_2_. DF =degrees of freedom*

Although the model converged, the final Hessian matrix revealed at least one negative eigenvalue, indicating a violation of the second-order optimality conditions. This suggests that the model may be too complex even trying trureg and firo otions and increasing the number of iterations.

##### Final model

The selected model for the oxygen-haemoglobin dissociation curve was built without random effects and included the effect of PCO_2_ on **b** and **x0.** The initial guess for the parameters were: a=97.5043671, b_fix_=15.2059958, b_PCO2_=1, x0_fix_=19.6783884, x0_PCO2_=1, s2e=3. The convergence criterion was set at 0.000000000000001 and the maximum number of iterations was set at 10000.

$$HbO_{2} =a \cdot e^{{-e}^{\frac{-(PO_{2}- x0)}{b}}}$$

where

b=b_fix_+b_PCO2_ · PCO_2_

x0=x0_fix_+x0_PCO2_ · PCO_2_

| **Parameter** | **Estimate** | **Standard Error** | **DF** | **P** | **95% Confidence Limits** | |
| --- | --- | --- | --- | --- | --- | --- |
| **a** | 97.1399 | 0.1548 | 346 | <.0001 | 96.8354 | 97.4444 |
| **b_fix_** | 9.2308 | 0.2089 | 346 | <.0001 | 8.8199 | 9.6418 |
| **b_PCO2_** | 0.1062 | 0.003742 | 346 | <.0001 | 0.09889 | 0.1136 |
| **x0_fix_** | 11.1305 | 0.1659 | 346 | <.0001 | 10.8043 | 11.4568 |
| **x0_PCO2_** | 0.1793 | 0.003120 | 346 | <.0001 | 0.1732 | 0.1855 |
| **s2e** | 3.2723 | 0.2488 | 346 | <.0001 | 2.7830 | 3.7616 |

*Table S15: Estimated parameters for the final nonlinear mixed model with no random effects and with only b and x0 depending on PCO_2_. DF =degrees of freedom*

AIC = 1404.1

##### Comparison between individual fittings and nonlinear mixed model

*Figure S6 – Figure shows the comparison between the individual fittings (dashed grey lines) and the fitting with nonlinear mixed model using Gompertz curve (solid red line) at different nominal PCO_2_ values. Green dots represent experimental data points, the red dashed lines represent the reference at 100%*

*A – nominal PCO_2_= 10 mmHg*

*B – nominal PCO_2_= 20 mmHg*

*C – nominal PCO_2_= 50 mmHg*

*D – nominal PCO_2_= 70 mmHg*

*E – nominal PCO_2_= 90 mmHg*

##### Experimental data points and Gompertz nonlinear mixed model fit with confidence intervals of all subjects at each nominal PCO_2_ value

*Figure S7 – Figure shows experimental data points (green dots) and Gompertz nonlinear mixed model fit (dashed black line) with confidence intervals (light blue band) of all subjects at each nominal PCO_2_ value. The red dashed line represents the reference at 100%*

*A B*

*C D*

*E*

##### Estimated P50 values

The values of PO_2_ at which hemoglobin is 50% saturated (P50) at different PCO_2_ values estimated with the nonlinear mixed model are reported in table S16.

| **PCO_2_ (mmHg)** | **P50 (mmHg)** | **Standard Error** | **DF** | **P** | **95% Confidence Interval** | |
| --- | --- | --- | --- | --- | --- | --- |
| 5 | 16.0225 | 0.129 | 346 | <.0001 | 15.7689 | 16.2762 |
| 10 | 17.1366 | 0.1191 | 346 | <.0001 | 16.9022 | 17.3709 |
| 15 | 18.2506 | 0.11 | 346 | <.0001 | 18.0342 | 18.467 |
| 20 | 19.3646 | 0.1018 | 346 | <.0001 | 19.1643 | 19.5649 |
| 25 | 20.4786 | 0.0948 | 346 | <.0001 | 20.2922 | 20.6651 |
| 30 | 21.5926 | 0.0892 | 346 | <.0001 | 21.4172 | 21.7681 |
| 35 | 22.7067 | 0.08533 | 346 | <.0001 | 22.5388 | 22.8745 |
| 40 | 23.8207 | 0.08341 | 346 | <.0001 | 23.6566 | 23.9847 |
| 45 | 24.9347 | 0.08358 | 346 | <.0001 | 24.7703 | 25.0991 |
| 50 | 26.0487 | 0.08583 | 346 | <.0001 | 25.8799 | 26.2176 |
| 55 | 27.1627 | 0.09001 | 346 | <.0001 | 26.9857 | 27.3398 |
| 60 | 28.2768 | 0.09587 | 346 | <.0001 | 28.0882 | 28.4653 |
| 65 | 29.3908 | 0.1031 | 346 | <.0001 | 29.188 | 29.5936 |
| 70 | 30.5048 | 0.1115 | 346 | <.0001 | 30.2856 | 30.724 |
| 75 | 31.6188 | 0.1207 | 346 | <.0001 | 31.3814 | 31.8563 |
| 80 | 32.7329 | 0.1307 | 346 | <.0001 | 32.4759 | 32.9898 |
| 85 | 33.8469 | 0.1412 | 346 | <.0001 | 33.5692 | 34.1245 |
| 90 | 34.9609 | 0.1521 | 346 | <.0001 | 34.6618 | 35.26 |
| 95 | 36.0749 | 0.1634 | 346 | <.0001 | 35.7536 | 36.3962 |
| 100 | 37.1889 | 0.1749 | 346 | <.0001 | 36.8449 | 37.533 |

*Table S16: P50 at different PCO_2_ values estimated with the model. DF =degrees of freedom*

As shown, at increasing PCO_2_ values, P50 is shifted to the right according to the equation:

P50 = 14.9085 + 0.2228 · PCO_2_ (P<0.0001)

*Figure S8 – Estimated P50 values at different PCO_2_ (light blue dots) and linear regression (grey line)*

##### Estimated HbO_2_ values for comparison with Bohr paper

| **PO_2_ (mmHg)** | **Estimated HbO_2_ at 5 mmHg PCO_2_** | **Estimated HbO_2_ at 10 mmHg PCO_2_** | **Estimated HbO_2_ at 20 mmHg PCO_2_** | **Estimated HbO_2_ at 40 mmHg PCO_2_** | **Estimated HbO_2_ at 80 mmHg PCO_2_** |
| --- | --- | --- | --- | --- | --- |
| 5 | 12.45 ± 0.67 [11.14 - 13.77] | 11.21 ± 0.57 [10.09 - 12.33] | 9.24 ± 0.42  [8.42 - 10.05] | 6.64 ± 0.24  [6.17 - 7.12] | 4.07 ± 0.22  [3.64 - 4.49] |
| 10 | 28.37 ± 0.64 [27.12 - 29.62] | 25.73 ± 0.58 [24.6 - 26.86] | 21.35 ± 0.46 [20.45 - 22.26] | 15.25 ± 0.3 [14.66 - 15.84] | 8.87 ± 0.3  [8.27 - 9.46] |
| 15 | 46.46 ± 0.46 [45.56 - 47.37] | 42.9 ± 0.43 [42.05 - 43.74] | 36.63 ± 0.37 [35.9 - 37.35] | 27.07 ± 0.28 [26.52 - 27.62] | 15.97 ± 0.34 [15.3 - 16.64] |
| 20 | 62.44 ± 0.41 [61.63 - 63.26] | 58.75 ± 0.36 [58.04 - 59.46] | 51.84 ± 0.29 [51.26 - 52.42] | 40.22 ± 0.23 [39.77 - 40.67] | 24.89 ± 0.33 [24.23 - 25.54] |
| 25 | 74.54 ± 0.42 [73.72 - 75.37] | 71.29 ± 0.37 [70.57 - 72.02] | 64.83 ± 0.28 [64.27 - 65.39] | 52.86 ± 0.2 [52.46 - 53.26] | 34.78 ± 0.29 [34.2 - 35.35] |
| 30 | 82.89 ± 0.38 [82.13 - 83.65] | 80.3 ± 0.35 [79.61 - 80.99] | 74.88 ± 0.28 [74.32 - 75.43] | 63.83 ± 0.2 [63.44 - 64.22] | 44.76 ± 0.26 [44.26 - 45.27] |
| 35 | 88.33 ± 0.32 [87.71 - 88.96] | 86.41 ± 0.3 [85.81 - 87] | 82.15 ± 0.26 [81.64 - 82.66] | 72.7 ± 0.2 [72.31 - 73.09] | 54.15 ± 0.24 [53.68 - 54.62] |
| 40 | 91.76 ± 0.25 [91.27 - 92.25] | 90.39 ± 0.24 [89.91 - 90.87] | 87.2 ± 0.22 [86.76 - 87.64] | 79.53 ± 0.19 [79.17 - 79.9] | 62.51 ± 0.24 [62.04 - 62.98] |
| 45 | 93.88 ± 0.19 [93.5 - 94.26] | 92.93 ± 0.2 [92.54 - 93.31] | 90.62 ± 0.19 [90.25 - 90.99] | 84.62 ± 0.17 [84.29 - 84.94] | 69.66 ± 0.24 [69.19 - 70.13] |
| 50 | 95.17 ± 0.16 [94.86 - 95.49] | 94.53 ± 0.16 [94.21 - 94.85] | 92.89 ± 0.16 [92.58 - 93.2] | 88.31 ± 0.14 [88.03 - 88.6] | 75.59 ± 0.23 [75.14 - 76.05] |
| 55 | 95.96 ± 0.15 [95.67 - 96.25] | 95.52 ± 0.15 [95.24 - 95.81] | 94.38 ± 0.14 [94.11 - 94.66] | 90.96 ± 0.13 [90.71 - 91.21] | 80.4 ± 0.22 [79.96 - 80.83] |
| 60 | 96.43 ± 0.14 [96.15 - 96.71] | 96.14 ± 0.14 [95.87 - 96.42] | 95.36 ± 0.13 [95.09 - 95.62] | 92.83 ± 0.12 [92.59 - 93.07] | 84.22 ± 0.2 [83.82 - 84.62] |
| 65 | 96.71 ± 0.14 [96.43 - 97] | 96.53 ± 0.14 [96.25 - 96.8] | 95.99 ± 0.13 [95.72 - 96.25] | 94.15 ± 0.12 [93.91 - 94.38] | 87.23 ± 0.19 [86.86 - 87.59] |
| 70 | 96.88 ± 0.15 [96.6 - 97.17] | 96.76 ± 0.14 [96.48 - 97.04] | 96.4 ± 0.14 [96.13 - 96.67] | 95.06 ± 0.12 [94.82 - 95.3] | 89.57 ± 0.17 [89.24 - 89.9] |
| 75 | 96.99 ± 0.15 [96.69 - 97.28] | 96.91 ± 0.15 [96.62 - 97.2] | 96.66 ± 0.14 [96.38 - 96.94] | 95.7 ± 0.13 [95.45 - 95.95] | 91.37 ± 0.15 [91.07 - 91.67] |
| 80 | 97.05 ± 0.15 [96.75 - 97.34] | 97.00 ± 0.15 [96.7 - 97.29] | 96.83 ± 0.14 [96.55 - 97.12] | 96.15 ± 0.13 [95.88 - 96.41] | 92.76 ± 0.14 [92.47 - 93.04] |
| 85 | 97.08 ± 0.15 [96.79 - 97.38] | 97.05 ± 0.15 [96.75 - 97.35] | 96.94 ± 0.15 [96.65 - 97.23] | 96.45 ± 0.14 [96.18 - 96.72] | 93.81 ± 0.14 [93.54 - 94.09] |
| 90 | 97.11 ± 0.15 [96.81 - 97.41] | 97.09 ± 0.15 [96.79 - 97.38] | 97.01 ± 0.15 [96.72 - 97.31] | 96.67 ± 0.14 [96.39 - 96.94] | 94.62 ± 0.14 [94.36 - 94.89] |
| 95 | 97.12 ± 0.15 [96.82 - 97.42] | 97.11 ± 0.15 [96.81 - 97.41] | 97.06 ± 0.15 [96.76 - 97.35] | 96.81 ± 0.14 [96.53 - 97.1] | 95.23 ± 0.13 [94.97 - 95.5] |
| 100 | 97.13 ± 0.15 [96.83 - 97.43] | 97.12 ± 0.15 [96.82 - 97.42] | 97.09 ± 0.15 [96.79 - 97.39] | 96.91 ± 0.15 [96.62 - 97.2] | 95.7 ± 0.14 [95.43 - 95.97] |
| 105 | 97.13 ± 0.15 [96.83 - 97.44] | 97.13 ± 0.15 [96.82 - 97.43] | 97.11 ± 0.15 [96.8 - 97.41] | 96.98 ± 0.15 [96.69 - 97.28] | 96.05 ± 0.14 [95.78 - 96.32] |
| 110 | 97.14 ± 0.15 [96.83 - 97.44] | 97.13 ± 0.15 [96.83 - 97.44] | 97.12 ± 0.15 [96.82 - 97.42] | 97.03 ± 0.15 [96.74 - 97.33] | 96.32 ± 0.14 [96.04 - 96.59] |
| 115 | 97.14 ± 0.15 [96.83 - 97.44] | 97.14 ± 0.15 [96.83 - 97.44] | 97.13 ± 0.15 [96.82 - 97.43] | 97.07 ± 0.15 [96.77 - 97.36] | 96.52 ± 0.14 [96.24 - 96.8] |
| 120 | 97.14 ± 0.15 [96.83 - 97.44] | 97.14 ± 0.15 [96.83 - 97.44] | 97.13 ± 0.15 [96.83 - 97.43] | 97.09 ± 0.15 [96.79 - 97.39] | 96.67 ± 0.14 [96.39 - 96.96] |
| 125 | 97.14 ± 0.15 [96.83 - 97.44] | 97.14 ± 0.15 [96.83 - 97.44] | 97.13 ± 0.15 [96.83 - 97.44] | 97.1 ± 0.15 [96.8 - 97.41] | 96.79 ± 0.15 [96.5 - 97.07] |
| 130 | 97.14 ± 0.15 [96.84 - 97.44] | 97.14 ± 0.15 [96.83 - 97.44] | 97.14 ± 0.15 [96.83 - 97.44] | 97.12 ± 0.15 [96.81 - 97.42] | 96.87 ± 0.15 [96.58 - 97.16] |
| 135 | 97.14 ± 0.15 [96.84 - 97.44] | 97.14 ± 0.15 [96.83 - 97.44] | 97.14 ± 0.15 [96.83 - 97.44] | 97.12 ± 0.15 [96.82 - 97.43] | 96.94 ± 0.15 [96.64 - 97.23] |
| 140 | 97.14 ± 0.15 [96.84 - 97.44] | 97.14 ± 0.15 [96.84 - 97.44] | 97.14 ± 0.15 [96.83 - 97.44] | 97.13 ± 0.15 [96.82 - 97.43] | 96.99 ± 0.15 [96.69 - 97.28] |
| 145 | 97.14 ± 0.15 [96.84 - 97.44] | 97.14 ± 0.15 [96.84 - 97.44] | 97.14 ± 0.15 [96.83 - 97.44] | 97.13 ± 0.15 [96.83 - 97.44] | 97.03 ± 0.15 [96.73 - 97.32] |
| 150 | 97.14 ± 0.15 [96.84 - 97.44] | 97.14 ± 0.15 [96.84 - 97.44] | 97.14 ± 0.15 [96.83 - 97.44] | 97.13 ± 0.15 [96.83 - 97.44] | 97.05 ± 0.15 [96.75 - 97.35] |

##### *Table S17: Estimated ± standard error [95% CI] HbO_2_ values at different nominal PCO_2_ values computed using the model.*

|  | **Percentage of oxygen uptake** | | | | |
| --- | --- | --- | --- | --- | --- |
| **PO_2_ (mmHg)** | **5 mmHg PCO_2_** | **10 mmHg PCO_2_** | **20 mmHg PCO_2_** | **40 mmHg PCO_2_** | **80 mmHg PCO_2_** |
| 5 | 11 | 7.5 | 5 | 3 | 1.5 |
| 10 | 28.5 | 20.5 | 14 | 9 | 4 |
| 15 | 51 | 36 | 27 | 18.5 | 8 |
| 20 | 67.5 | 54 | 41 | 29.5 | 14 |
| 25 | 76 | 67 | 54 | 40 | 22 |
| 30 | 82 | 74.5 | 63.5 | 50 | 31 |
| 35 | 86 | 79.5 | 71 | 58 | 40 |
| 40 | 89 | 84 | 77 | 66.5 | 49 |
| 45 | 91 | 87.5 | 82 | 73 | 56 |
| 50 | 92.5 | 90 | 86 | 78.5 | 62.5 |
| 60 | 95 | 93.5 | 90.5 | 86 | 73 |
| 70 | 97 | 95.5 | 94 | 91 | 80.5 |
| 80 | 98 | 97 | 96 | 94.5 | 87 |
| 90 | 98.5 | 98 | 97 | 96 | 91.5 |
| 100 | 99 | 98.5 | 98 | 97 | 95 |
| 150 | 100 | 100 | 100 | 99.8 | 99.5 |

##### *Table S18: Percentage of oxygen uptake from Bohr et. al. (Bohr C, Hasselbalch K, Krogh A. Ueber einen in biologischer Beziehung wichtigen Einfluss, den die Kohlensäurespannung des Blutes auf dessen Sauerstoffbindung übt. Skandinavisches Archiv Für Physiologie (1904) 16(2):402-412.)*

##### *Figure S9 – Comparison of oxygen-haemoglobin dissociation curves estimated by our model (blue) and percentages of oxygen uptake reported by Bohr et al. (red) at different PCO_2_ values. (Bohr C, Hasselbalch K, Krogh A. Ueber einen in biologischer Beziehung wichtigen Einfluss, den die Kohlensäurespannung des Blutes auf dessen Sauerstoffbindung übt. . Skandinavisches Archiv Für Physiologie (1904) 16(2):402-412.)*

## Electrolytes

### Collinearity

The collinearity diagnostics revealed that all condition indices were below the threshold of 30 and VIF (collinearity between PCO_2_ and HbO_2_) is equal to 1.06, indicating no severe collinearity issues among the independent variables.

| **Collinearity Diagnostics** | | | | | |
| --- | --- | --- | --- | --- | --- |
| **Number** | **Eigenvalue** | **Condition Index** | **Proportion of Variation** | | |
|  |  |  | **Intercept** | **HbO_2_** | **PCO_2_** |
| **1** | 2.69130 | 1.00000 | 0.01000 | 0.01442 | 0.03072 |
| **2** | 0.26205 | 3.20471 | 0.00993 | 0.14638 | 0.64953 |
| **3** | 0.04665 | 7.59578 | 0.98007 | 0.83921 | 0.31975 |

##### *Table S19: Collinearity diagnostics.*

### SID

The preliminary graphical analyses of the relationships between SID and HbO_2_ at both nominal and at 5-steps PCO_2_ levels (e.g., the group centered at 5 mmHg included values from 2.5 to 7.5 mmHg, the group at 10 mmHg included values from 7.5 to 12.5 mmHg, and so on, up to 110 mmHg) suggested a linear association between the variables. Additionally, the preliminary graphical analyses of the relationships between SID and PCO_2_ at both nominal PCO_2_ and at 5-steps HbO_2_ classes (5% intervals from 0% to 100%, centered on multiples of 5 %) suggested a quadratic association between the variables.

*Figure S10 – Graphical analyses of the relationships between SID and HbO2 at nominal PCO_2_ values (different colours). Dots represent experimental data points; dashed lines represent linear regression. Left panel: all subjects. Right panel: single subject representation*

*Figure S11 – Graphical analyses of the relationships between SID and PCO_2_ at nominal PCO_2_ values (different colours). Dots represent experimental data points, dashed lines represent 2^nd^ order regressions. Left panel: all subjects. Right panel: single subject representation*

*Figure S12 – Graphical analyses of the relationships between SID and HbO2 at 5-steps PCO_2_ values (different colours). Dots represent experimental data points; dashed lines represent linear regression. Left panel: all subjects. Right panel: single subject representation*

*Figure S13 – Graphical analyses of the relationships between SID and PCO_2_ at 5-steps PCO_2_ values (different colours). Dots represent experimental data points, dashed lines represent 2^nd^ order regressions. Left panel: all subjects. Right panel: single subject representation*

Details on SID model selection are reported in the table S20.

| ***Overall model parameters*** |  |  |  |  |  |
| --- | --- | --- | --- | --- | --- |
| **Number of observations used** | 346 |  |  |  |  |
| **Number of observations read** | 346 |  |  |  |  |
| **Missing values** | 0 |  |  |  |  |
| **Random intercept group variable: patients** | 6 |  |  |  |  |
| **Random slope variable: HbO_2_ and PCO_2_** |  |  |  |  |  |
| **Observations per patients (max):** | 76 |  |  |  |  |
| ***Model selection*** | **P** |  |  |  |  |
| ***Random HbO_2_ effect (assessed on null model)*** |  |  |  |  |  |
| **Random intercept vs. standard linear regression model** | 0.5691 |  |  |  |  |
| **Random slope vs. random intercept model** | NA |  |  |  |  |
| ***Random PCO_2_ effect (assessed on null model)*** |  |  |  |  |  |
| ***with HbO_2_ among fixed effects*** |  |  |  |  |  |
| **Random intercept vs. standard linear regression model** | <.0001 |  |  |  |  |
| **Random slope PCO_2_ vs. random intercept model** | 0.0002 |  |  |  |  |
| ***Functional form of association between SID and PCO_2_*** |  |  |  |  |  |
| **Linear vs. quadratic** | <.0001 |  |  |  |  |
| ***Interaction between HbO_2_ and PCO_2_ vs. no interaction*** | 0.0001 |  |  |  |  |
| **Fixed effect parameters** | **Coeff.** | **std err** | **95% CI** | | **P** |
| **Intercept** | 31.5013 | 0.5911 | 30.0517 | 32.9509 | <.0001 |
| **HbO_2_: linear** | -0.0327 | 0.0015 | -0.0357 | -0.0297 | <.0001 |
| **PCO_2_: linear** | 0.3763 | 0.0085 | 0.3584 | 0.3943 | <.0001 |
| **PCO_2_: quadratic** | -0.0017 | 0.0001 | -0.0018 | -0.0016 | <.0001 |
| **~~HbO~~_~~2~~_ ~~and PCO~~_~~2~~_ ~~interaction~~**  **Removed from the model as it was not statistically significant** |  |  |  |  |  |
| **Random effect parameters** | **Coeff.** | **std err** | **95% CI** | | **P** |
| **Random intercept** | 1.8827 | 1.2158 | -0.5004 | 4.2657 | 0.0608 |
| **Unstructured covariance** | -0.0173 | 0.0123 | -0.0414 | 0.0069 | 0.1609 |
| **Random slope** | 0.0002 | 0.0001 | -0.0001 | 0.0005 | 0.0666 |
| **Residual variance** | 0.6074 | 0.0472 | 0.5150 | 0.6998 | <.0001 |

*Table S20: Details on SID model selection. NA: not applicable; Coeff. = coefficients; std err = standard error*

| **Covariance matrix** | **AIC (Akaike Information Criterion)** |
| --- | --- |
| UN (Unstructured) | 892.8 |
| CS (Compound Symmetry) | 1055.3 |
| AR(1) (Autoregressive) | 1055.3 |
| TOEP (Toeplitz) | 935.3 |
| VC (Variance Components) | 897.0 |

*Table 21: AIC values according to the covariance matrix applied*

*Figure S14 - Residuals distribution (left panel) and QQ plot (right panel) for SID distribution.*

*Figure S15 – Conditioned residuals distribution (left panel) and QQ plot (right panel) for SID distribution.*

*Figure S16 - Graphical representation of the model results according to HbO_2_ at different PCO_2_ (continuous variables) and vice versa.*

The final polynomial multilevel model included HbO_2_ and PCO_2_ as predictors of both the intercept and the linear terms, as well as a quadratic term for PCO_2_. Random intercept at the subject level and slope for PCO_2_ were included. The model was implemented with a completely general (unstructured) covariance matrix (AIC 892.8). As HbO_2_ increases, SID decreases linearly, as indicated by the negative coefficient (Coeff. = -0.0327, p < .0001). Conversely, as PCO_2_ increases, SID initially increases due to the positive linear coefficient (Coeff. = 0.3763, p < .0001), but this effect diminishes at higher levels of PCO_2_, as shown by the negative quadratic coefficient (Coeff. = -0.0017, p < .0001). Interaction was removed from the model as not statistically significant.

*Figure S17 - Relationship between estimated SID and HbO_2_ at different PCO_2_ (5 to 100 by 5 mmHg) and between SID and PCO_2_ and different HbO_2_ (0% to 100% by 5%). Bands represent 95% confidence limits.*

*Figure S18 - Relationship between estimated SID and HbO_2_ at different PCO_2_ (5 to 110 by 5 mmHg) and between SID and PCO_2_ and different HbO_2_ (0% to 100% by 5%).*

*Figure S19 - Relationship between estimated SID and HbO_2_ at different PCO_2_ (5 to 110 by 5 mmHg).* *Dots represent experimental points and lines connects data from different subjects.*

*Figure S20 - Relationships between estimated SID and PCO_2_ at different HbO_2_ (0% to 100% by 5% mmHg). Dots represent experimental points and lines connects data from different subjects.*

The results of the bootstrap analysis, based on 1000 resamples, are reported in the table below. These results indicate precise and reliable estimates for the model parameters.

| **Effect** | **N** | **Mean** | **SD** | **LCL** | **UCL** |
| --- | --- | --- | --- | --- | --- |
| **Intercept** | 1000 | 31.4190 | 0.2153 | 30.7184 | 32.0435 |
| **HbO_2_** | 1000 | -0.0329 | 0.0015 | -0.0372 | -0.0277 |
| **PCO_2_: quadratic** | 1000 | -0.0017 | 0.00007 | -0.0020 | -0.0015 |
| **PCO_2_: linear** | 1000 | 0.3784 | 0.0070 | 0.3513 | 0.4036 |

*Table S22 - Bootstrap analysis. Parameters are expressed as Mean ± standard deviation (SD) and lower and upper confidence interval (LCL and UCL, respectively)*

### Chloride

The preliminary graphical analyses of the relationships between Cl^-^ and HbO_2_ at both nominal and at 5-steps PCO_2_ levels suggested a linear association between the variables. Additionally, the preliminary graphical analyses of the relationships between Cl^-^ and PCO_2_ at both nominal PCO_2_ and at 5-steps HbO_2_ classes suggested a quadratic association between the variables.

*Figure S21 – Graphical analyses of the relationships between Cl^-^ and HbO^2^ at nominal PCO_2_ values (different colours). Dots represent experimental data points, dashed lines represent linear regression. Left panel: all subjects. Right panel: single subject representation*

*Figure S22– Graphical analyses of the relationships between Cl^-^ and PCO_2_ at nominal PCO_2_ values (different colours). Dots represent experimental data points, dashed lines represent 2^nd^ order regressions. Left panel: all subjects. Right panel: single subject representation*

*Figure S23 – Graphical analyses of the relationships between Cl^-^ and HbO2 at 5-steps PCO_2_ values (different colours). Dots represent experimental data points; dashed lines represent linear regression. Left panel: all subjects. Right panel: single subject representation*

*Figure S24 – Graphical analyses of the relationships between Cl^-^ and PCO_2_ at 5-steps PCO_2_ values (different colours). Dots represent experimental data points, dashed lines represent 2^nd^ order regressions. Left panel: all subjects. Right panel: single subject representation*

Details on the model selection are reported in table S23:

| ***Overall model parameters*** |  |  |  |  |  |
| --- | --- | --- | --- | --- | --- |
| **Number of observation used** | 346 |  |  |  |  |
| **Number of observation read** | 346 |  |  |  |  |
| **Missing values** | 0 |  |  |  |  |
| **Random intercept group variable: patients** | 6 |  |  |  |  |
| **Random slope variable: HbO_2_ and PCO_2_** |  |  |  |  |  |
| **Observations per patients (max):** | 76 |  |  |  |  |
| ***Model selection*** | **P** |  |  |  |  |
| ***Random HbO_2_ effect (assessed on null model)*** |  |  |  |  |  |
| **Random intercept vs. standard linear regression model** | 0.0008 |  |  |  |  |
| **Random slope vs. random intercept model** | 0.8925 |  |  |  |  |
| ***Random PCO_2_ effect (assessed on null model)*** |  |  |  |  |  |
| ***with HbO_2_ among fixed effects*** |  |  |  |  |  |
| **Random intercept vs. standard linear regression model** | <.0001 |  |  |  |  |
| **Random slope PCO_2_ vs. random intercept model** | 0.0016 |  |  |  |  |
| ***Functional form of association between SID and PCO_2_*** |  |  |  |  |  |
| **Linear vs. quadratic** | <.0001 |  |  |  |  |
| ***Interaction between HbO_2_ and PCO_2_ vs. no interaction*** | 0.2287 |  |  |  |  |
| **Fixed effect parameters** | **Coeff.** | **std err** | **95% CI** | | **P** |
| **Intercept** | 111.0100 | 0.5846 | 109.5700 | 112.4500 | <.0001 |
| **HbO_2_: linear** | 0.0242 | 0.0014 | 0.0214 | 0.0271 | <.0001 |
| **PCO_2_: linear** | -0.2438 | 0.0068 | -0.2575 | -0.2302 | <.0001 |
| **PCO_2_: quadratic** | 0.0012 | 0.0001 | 0.0011 | 0.0014 | <.0001 |
| **~~HbO~~_~~2~~_ ~~and PCO~~_~~2~~_ ~~interaction~~** |  |  |  |  |  |
| **Random effect parameters** | **Coeff.** | **std err** | **95% CI** | | **P** |
| **Random intercept** | 1.8647 | 1.2003 | -0.4879 | 4.2173 | 0.0601 |
| **Unstructured covariance** | -0.0074 | 0.0069 | -0.0209 | 0.0061 | 0.2823 |
| **Random slope** | 0.0001 | 0.0001 | 0.0000 | 0.0002 | 0.0750 |
| **Residual variance** | 0.5281 | 0.0410 | 0.4478 | 0.6084 | <.0001 |

*Table S23: Details on Cl^-^ model selection. NA: not applicable; Coeff. = coefficients; std err = standard error*

| **Covariance matrix** | **AIC (Akaike Information Criterion)** |
| --- | --- |
| UN (Unstructured) | 846.0 |
| CS (Compound Symmetry) | 1031.9 |
| AR(1) (Autoregressive) | 1031.9 |
| TOEP (Toeplitz) | 888.7 |
| VC (Variance Components) | 846.0 |

*Table S24: AIC values according to the covariance matrix applied*

*Figure S25 - Residuals distribution (left panel) and QQ plot (right panel).*

*Figure S26 – Conditioned residuals distribution (left panel) and QQ plot (right panel).*

*Figure S27 – Graphical representation of the model results according to the HbO_2_ at different PCO_2_ (continuous variables) and vice versa*

The final polynomial multilevel model included HbO_2_ and PCO_2_ as predictors of both the intercept and the linear terms, as well as a quadratic term for PCO_2_. Random intercept at the subject level and slope for PCO_2_ were included. The model was implemented with a completely general (unstructured) covariance matrix (AIC 846.0). As HbO_2_ increases, Cl^-^ increases linearly, as indicated by the positive coefficient (Coeff. = 0.0242, p < .0001). Conversely, as PCO_2_ increases, Cl^-^ linearly decreases due to the negative linear coefficient (Coeff. = -0.2438, p < .0001), but this effect diminishes at higher PCO_2_ levels as shown by the positive quadratic coefficient (Coeff. = -0.0012, p < .0001).

*Figure S28 - Relationship between estimated Cl^-^ and HbO_2_ at different PCO_2_ (5 to 100 by 5 mmHg) and between Cl^-^ and PCO_2_ and different HbO_2_ (0% to 100% by 5%). Bands represent 95% confidence limits.*

*Figure S29 - Relationships between estimated Cl^-^ and HbO_2_ at different PCO_2_ (5 to 100 by 5 mmHg) and between Cl^-^ and PCO_2_ and different HbO_2_ (0% to 100% by 5%).*

*Figure S30 - Relationship between estimated Cl^-^ and HbO_2_ at different PCO_2_ (5 to 110 mmHg by 5 mmHg). Dots represent experimental points and lines connects data from different subjects*

*Figure S31 - Relationship between estimated Cl^-^ and PCO_2_ at different HbO_2_ (5 to 100 mmHg by 5 mmHg). Dots represent experimental points and lines connects data from different subjects*

The results of the bootstrap analysis, based on 1000 resamples, are reported in the table below. These results indicate precise and reliable estimates for the model parameters.

| **Effect** | **N** | **Mean** | **DS** | **LCL** | **UCL** |
| --- | --- | --- | --- | --- | --- |
| **Intercept** | 1000 | 111.0170636 | 0.2020519 | 110.3900678 | 111.7003548 |
| **HbO_2_** | 1000 | 0.0241653 | 0.0015104 | 0.0190333 | 0.0296100 |
| **PCO_2_: quadratic** | 1000 | 0.0012522 | 0.000051948 | 0.0010765 | 0.0014285 |
| **PCO_2_: linear** | 1000 | -0.2440293 | 0.0057700 | -0.2627084 | -0.2264551 |

*Table S25 - Bootstrap analysis. Parameters are expressed as Mean ± standard deviation (SD) and lower and upper confidence interval (LCL and UCL, respectively)*

### Sodium

The preliminary graphical analyses of the relationships between Na^+^ and HbO_2_ at both nominal and at 5-steps PCO_2_ levels suggested a linear association between the variables. Additionally, the preliminary graphical analyses of the relationships between Na^+^ and PCO_2_ at both “nominal” PCO_2_ and at 5-steps HbO_2_ classes suggested a quadratic association between the variables.

*Figure S32 – Graphical analyses of the relationships between Na^+^ and HbO2 at nominal PCO_2_ values (different colours). Dots represent experimental data points, dashed lines represent linear regression. Left panel: all subjects. Right panel: single subject representation*

*Figure S33 – Graphical analyses of the relationships between Na^+^ and PCO_2_ at nominal PCO_2_ values (different colours). Dots represent experimental data points, dashed lines represent 2^nd^ order regressions. Left panel: all subjects. Right panel: single subject representation*

*Figure S34 – Graphical analyses of the relationships between Na^+^ and HbO2 at 5-steps PCO_2_ values (different colours). Dots represent experimental data points; dashed lines represent linear regression. Left panel: all subjects. Right panel: single subject representation*

*Figure S35 – Graphical analyses of the relationships between Na^+^ and PCO_2_ at 5-steps PCO_2_ values (different colours). Dots represent experimental data points, dashed lines represent 2^nd^ order regressions. Left panel: all subjects. Right panel: single subject representation*

Details on the model selection are reported in table S26

| ***Overall model parameters*** |  |  |  |  |  |
| --- | --- | --- | --- | --- | --- |
| **Number of observation used** | 346 |  |  |  |  |
| **Number of observation read** | 346 |  |  |  |  |
| **Missing values** | 0 |  |  |  |  |
| **Random intercept group variable: patients** | 6 |  |  |  |  |
| **Random slope variable: HbO_2_ and PCO_2_** |  |  |  |  |  |
| **Observations per patients (max):** | 76 |  |  |  |  |
| ***Model selection*** | **P** |  |  |  |  |
| ***Random HbO_2_ effect (assessed on null model)*** |  |  |  |  |  |
| **Random intercept vs. standard linear regression model** | <.0001 |  |  |  |  |
| **Random slope vs. random intercept model** | 0.0269 |  |  |  |  |
| ***Random PCO_2_ effect (assessed on null model)*** |  |  |  |  |  |
| ***with HbO_2_ among fixed and random effects*** |  |  |  |  |  |
| **~~Random intercept vs. standard linear regression model~~** |  |  |  |  |  |
| **Random intercept model + random slope HbO_2_ + random slope PCO_2_ vs. random intercept model + random slope HbO_2_** | <.0001 |  |  |  |  |
| ***Functional form of association between SID and PCO_2_*** |  |  |  |  |  |
| **Linear vs. quadratic** | <.0001 |  |  |  |  |
| ***Interaction between HbO_2_ and PCO_2_ vs. no interaction*** | 0.0009 |  |  |  |  |
| **Fixed effect parameters** | **Coeff.** | **Std err** | **95% CI** | | **P** |
| **Intercept** | 137.740 | 0.817 | 135.810 | 139.670 | <.0001 |
| **HbO_2_: linear*** | 0.000072 | 0.003948 | -0.007780 | 0.007919 | 0.986 |
| **PCO_2_: linear** | 0.1339 | 0.0100 | 0.1138 | 0.1540 | <.0001 |
| **PCO_2_: quadratic** | -0.000500 | 0.000059 | -0.000620 | -0.000380 | <.0001 |
| **HbO_2_ and PCO_2_ interaction** | -0.000130 | 0.000060 | -0.000250 | -0.000010 | 0.030 |
| **Random effect parameters** | **Coeff.** | **std err** | **95% CI** | | **P** |
| **UN(1,1)** | 3.2228 | 2.1310 | -0.9541 | 7.3996 | 0.0652 |
| **UN(2,1)** | -0.0062 | 0.0048 | -0.0157 | 0.0033 | 0.2016 |
| **UN(2,2)** | 0.0000 | 0.0000 | 0.0000 | 0.0000 | 0.3644 |
| **UN(3,1)** | -0.0191 | 0.0144 | -0.0473 | 0.0090 | 0.1822 |
| **UN(3,2)** | 0.0000 | 0.0000 | 0.0000 | 0.0001 | 0.2419 |
| **UN(3,3)** | 0.0002 | 0.0001 | -0.0001 | 0.0004 | 0.0716 |
| **Residual variance** | 0.6674 | 0.0524 | 0.5648 | 0.7701 | <.0001 |

*Table S26: Details on Na^+^ model selection. Coeff. = coefficients; std err = standard error; *not statistically significant*

| **Covariance matrix** | **AIC (Akaike Information Criterion)** |
| --- | --- |
| UN (Unstructured) | 940.9 |
| CS (Compound Symmetry) | 1028.6 |
| AR(1) (Autoregressive) | 1028.6 |
| TOEP (Toeplitz) | 1029.5 |
| VC (Variance Components) | 945.1 |

*Table S27: AIC values according to the covariance matrix applied*

*Figure S36 - Residuals distribution (left panel) and QQ plot (right panel).*

*Figure S37 - Conditioned residuals distribution (left panel) and QQ plot (right panel).*


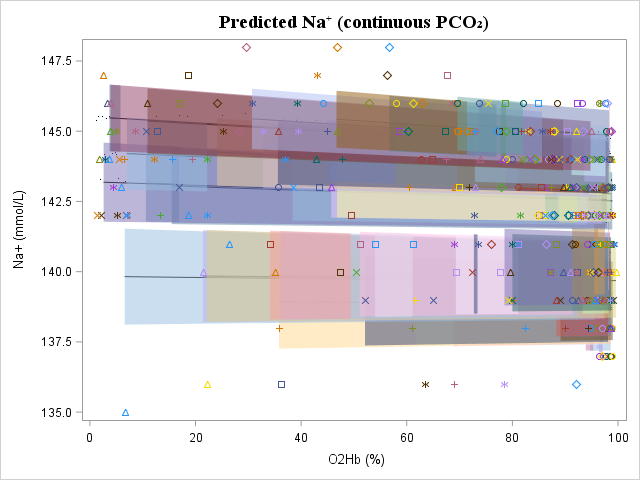

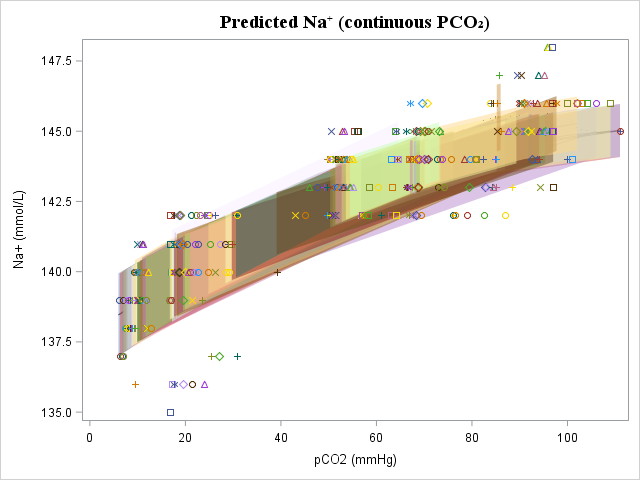


*Figure S38 - Graphical representation of the model results according to the HbO_2_ at different PCO_2_ (continuous variables) and vice versa*

The final polynomial multilevel model included HbO_2_ and PCO_2_ as predictors of both the intercept and the linear terms, as well as a quadratic term for PCO_2_. The interaction between the independent variables was also included in the model. Random intercept at the subject level and slope for HbO_2_ and PCO_2_ were included. The model was implemented with a completely general (unstructured) covariance matrix (AIC 940.9). As PCO_2_ increases, Na^+^ initially increases due to the positive linear coefficient (Coeff. = 0.134, p < .0001), but this effect diminishes at higher levels of PCO_2_, as shown by the negative quadratic coefficient (Coeff. = -0.00050, p < .0001). HbO_2_ linear effect is not statistically significant. Additionally, the interaction between HbO_2_ and PCO_2_ suggests a slight but significant modification of the SID trend (Coeff. = -0.0001, p = 0.030), indicating that HbO_2_ reduces SID, especially at higher levels of PCO_2_.

*Figure S39 - Relationship between estimated Na^+^ and HbO_2_ at different PCO_2_ (5 to 100 by 5 mmHg) and between Na^+^ and PCO_2_ and different HbO_2_ (0% to 100% by 5%). Bands represent 95% confidence limits.*

*Figure S40 - Relationships between estimated Na^+^ and HbO_2_ at different PCO_2_ (5 to 100 by 5 mmHg) and between Na^+-^ and PCO_2_ and different HbO_2_ (0% to 100% by 5%).*

*Figure S41 - Relationship between estimated Na^+^ and HbO_2_ at different PCO_2_ (5 to 110 mmHg by 5 mmHg). Dots represent experimental points and lines connects data from different subjects*

*Figure S42 - Relationship between estimated Na^+^ and PCO_2_ at different HbO_2_ (5 to 100 mmHg by 5 mmHg). Dots represent experimental points and lines connects data from different subjects*

The bootstrap analysis, performed on 730 steps, appear robust and reliable, suggesting that the model provides is stable and accurate.

| **Effect** | **N** | **Mean** | **SD** | **LCL** | **UCL** |
| --- | --- | --- | --- | --- | --- |
| **Intercept** | 730 | 137.8217 | 0.4352 | 136.2276 | 139.25267 |
| **HbO_2_** | 730 | -0.0005 | 0.0047 | -0.015 | 0.0167 |
| **HbO_2·_PCO_2_** | 730 | -0.0001 | 0.00008 | -0.0004 | 0.00008 |
| **PCO_2·_PCO_2_** | 730 | -0.0005 | 0.00006 | -0.0006 | -0.0002 |
| **PCO_2_** | 730 | 0.1313 | 0.0094 | 0.1044 | 0.1592 |

*Table S28 - Bootstrap analysis. Parameters are expressed as Mean ± standard deviation (SD) and lower and upper confidence interval (LCL and UCL, respectively)*

## Electrolytes model validation

Figures S43 and S44 present the comparison between our model and experimental data from 18 healthy subjects, as reported by Langer et al. (*J Appl Physiol (1985). 2021 Aug 1;131(2):464-473. doi: 10.1152/japplphysiol.00787.2020. Epub 2021 Jun 17.*)

Values of SID, Na⁺ and Cl⁻ were estimated using the polynomial mixed models at the average PCO_2_ values reported in the study and assuming HbO_2_ = 98% as the original paper did not report HbO₂ values. The authors stated that venous blood samples were tonometrically oxygenated at 21% O_2_. Based on this, we assumed that the blood was fully oxygenated at the end of the tonometry process. Since HbO_2_ is typically slightly lower than oxygen saturation due to the presence of carboxyhemoglobin and methemoglobin, we adopted a physiologically plausible HbO_2_ value of 98% for our estimations. Absolute values (figure S43) and the differences from the reference value at 2% CO_2_ (figure S44) were plotted against PCO_2_ to assess model performance across a range of respiratory conditions. The model showed good agreement with the average experimental data, particularly in terms of the differences from the reference condition, supporting its validity and robustness.

| **Variable** | **2% CO₂** | **5% CO₂** | **12% CO₂** | **20% CO₂** |
| --- | --- | --- | --- | --- |
| **PCO₂ (mmHg)** | 18.2 ± 2.1 | 32.1 ± 3.4 | 68.3 ± 4.9 | 124.4 ± 10.2 |
| **pH** | 7.61 ± 0.04 | 7.46 ± 0.03 | 7.24 ± 0.03 | 7.07 ± 0.03 |
| **HCO₃⁻ (mmol/L)** | 18.0 ± 1.2 | 22.8 ± 1.8 | 29.6 ± 1.6 | 35.7 ± 2.0 |
| **Na⁺ (mmol/L)** | 137.3 ± 2.1 | 138.9 ± 2.0 | 140.7 ± 2.1 | 142.7 ± 2.1 |
| **K⁺ (mmol/L)** | 4.23 ± 0.32 | 4.25 ± 0.36 | 4.32 ± 0.28 | 4.48 ± 0.39 |
| **Ca²⁺ (mmol/L)** | 1.09 ± 0.05 | 1.17 ± 0.05 | 1.28 ± 0.05 | 1.36 ± 0.06 |
| **Mg²⁺ (mmol/L)** | 2.10 ± 0.14 | 2.11 ± 0.15 | 2.11 ± 0.15 | 2.11 ± 0.15 |
| **Cl⁻ (mmol/L)** | 111 ± 2 | 109 ± 2 | 106 ± 2 | 104 ± 2 |
| **Lac⁻ (mmol/L)** | 1.9 ± 0.5 | 1.7 ± 0.6 | 1.7 ± 0.5 | 1.7 ± 0.5 |
| **SID (mmol/L)** | 32.8 ± 2.7 | 36.6 ± 2.2 | 41.6 ± 2.4 | 46.3 ± 2.9 |

*Table S29: whole blood tonometry data in 18 healthy subjects from Langer at al. Values are means ± SD.*

*Figure S43: comparison between experimental data from Langer et al. (mean ± SD, red dots) and values estimated using polynomial mixed models (green dots) for SID (left panel), Chloride (middle panel) and Sodium (right panel) at varying PCO_2_.*

*Figure S44:* *comparison between experimental data from Langer et al. (mean ± SD, red dots) and values estimated using polynomial mixed models (green dots) for SID variations (left panel), Chloride variations (middle panel) and Sodium variations (right panel) at varying PCO_2_.* *Values are expressed as difference from the first value at 2% CO_2_.*
